# Blinded by inequity: a scoping review of income-related and structural disparities in eye care in the United States

**DOI:** 10.3389/fpubh.2026.1885854

**Published:** 2026-10-19

**Authors:** Alexandra K. Lersten, Carrie B. Dolan

**Affiliations:** Department of Health Sciences, College of William & Mary, Williamsburg, VA, United States

**Keywords:** eye care, health disparities, healthcare access, multiple streams framework, socioeconomic factors

## Abstract

**Background:**

Visual impairment is a leading cause of preventable disability in the United States, despite the availability of effective screening, corrective, and surgical procedures. Lower household income has been associated with timely access to eye care, with low-income populations bearing a disproportionate burden.

**Objective:**

This scoping review characterizes evidence on income, socioeconomic, and structural barriers to eye care access in the United States, identifies interventions designed to address these barriers, and applies Kingdon’s Multiple Streams Framework to examine how problem recognition, policy solutions, and political factors shape progress toward equitable eye care.

**Methods:**

A scoping review was conducted following the Arksey and O’Malley methodological framework and PRISMA-ScR reporting guidelines. PubMed, Scopus, CINAHL, and Google Scholar were searched for studies published between 2001 and 2025 examining income-related disparities in U.S. eye care access. The review included 19 sources: 17 identified through database searches and two grey literature sources identified outside the search. Data were extracted, synthesized thematically, and interpreted using Kingdon’s Multiple Streams Framework.

**Results:**

Studies directly measuring income or poverty generally found that lower income was associated with reduced access to preventive and routine eye care and poorer vision outcomes. Across the broader evidence base, income-linked insurance measures and structural barriers, including inadequate coverage and limited availability of affordable services, were also associated with disparities in access. Community-based vision screening, integrated eye care within community health centers, and teleophthalmology were associated with improved access or service utilization in specific populations but were typically localized and resource-dependent. Application of Kingdon’s Multiple Streams Framework identified greater representation of the problem and policy streams, while politics-stream evidence was less frequently represented. Given the literature-identification strategy, this pattern may reflect characteristics of the included evidence base rather than an absence of political activity.

**Conclusion:**

Income-related disparities in eye care access are associated with structural and financial barriers linked to reduced access to timely services. Addressing these inequities may require greater alignment among recognized needs, promising interventions, and policy priorities, as well as enhanced integration of eye care within broader health systems. Establishing eye care as a more integrated component of health policy represents a potential approach to improving outcomes among low-income populations.

## Introduction

Low-income populations experience disproportionately limited access to eye care and face higher risks of vision impairment and related health consequences than their better-off counterparts. Evidence of these disparities is apparent across multiple measures of eye care access and vision health. For example, a 2013 analysis of the 2002–2008 National Health Interview Survey observed persistent socioeconomic disparities in eye care accessibility and usage (1). Nine percent of people reporting a need for glasses in 2017 also stated they were unable to afford them (2). Likewise, data from the National Health and Nutrition Examination Survey found that higher household income (>$75,000) was associated with lower odds of visual impairment (3). These disparities are also evident among children, with those in families below the federal poverty line twice as likely to have visual impairment as children in families with incomes at least 200% of the federal poverty level (4).

Financial barriers to eye care extend beyond household income to the structure and availability of insurance coverage. In many states, Medicaid treats eye care as an auxiliary rather than an essential health service. More than 6 million Medicaid enrollees reside in states lacking comprehensive vision exam coverage, and more than 14 million in states lacking coverage for eyeglasses (5). Even when coverage is available, however, access to providers may remain limited. One study demonstrated that obtaining an eye care appointment was more difficult for individuals with Medicaid than for those with private insurance (6).

Limitations within the health care safety net may create additional barriers for low-income and uninsured populations. Federally qualified health centers (FQHCs) serve as primary care sources for low-income individuals and often represent the only accessible entry point for care for uninsured populations. Nearly seven out of 10 FQHC patients have incomes at or below the federal poverty level, yet 69% of FQHCs do not provide on-site comprehensive eye exams (7). Together, gaps in affordability, insurance coverage, provider access, and safety-net services may contribute to greater reliance on emergency departments (EDs) for eye-related concerns among uninsured populations. Lack of insurance coverage is associated with emergent eye-related cases in the ED. Quintero et al. suggest that improving access to ophthalmologic care in uninsured communities may reduce the progression of untreated chronic eye disease to eye-related emergency department visits (8).

Beyond affordability, insurance coverage, and provider access, low-income populations may face additional barriers to obtaining eye care. For example, informational barriers may be associated with delayed care-seeking, as public awareness of common eye diseases remains low. An online nationwide poll of 2,044 US adults in 2014 found that 25% were unfamiliar with any eye conditions, 37.3% had never heard of diabetic retinopathy, and half did not know of the existence of macular degeneration (9). Health literacy may also be associated with engagement with care. Among patients with glaucoma, low health literacy has been associated with missed appointments and lower medication use (10). Geographic barriers further compound these challenges. A 2017 review that analyzed 2012 Medicare data found that cataract care recipients who resided in rural areas traveled further for ophthalmic care than urban and suburban residents (11). More broadly, variability in eye care service availability and provider distribution is associated with access to comprehensive eye care services across populations (12).

These barriers are particularly important because inadequate access to eye care may have consequences that extend beyond vision health. Visual impairment has been associated with depression, loss of productivity, injuries, and premature death (1). Vision impairment and socioeconomic disadvantage are also closely intertwined. Data from the 2002–2004 Medical Expenditure Panel Survey found that individuals with visual impairment were more than twice as likely to rely on Medicaid as individuals without visual impairment (13). Delayed or forgone eye care may also contribute to later detection and management of ocular disease. Observational studies have reported a higher prevalence of diabetic retinopathy among lower-income populations; for example, individuals with lower income levels report a greater prevalence of diabetic retinopathy than their higher-income counterparts (14).

In response to persistent gaps in access and utilization, a variety of policy and programmatic approaches have been developed. The National Academies of Sciences, Engineering, and Medicine have highlighted vision health as a population health priority (15). Additionally, nonprofit public health organizations, including Prevent Blindness America (PBA), have implemented vision screening programs targeting uninsured and vulnerable populations. A review assessing the effectiveness of PBA’s vision screening program found that the majority of uninsured adults in the program showed high rates of visual problems that required more comprehensive treatment, yet rates of follow-up with an eye care professional after referral remained consistently low (16). This gap in follow-up highlights a key barrier in linkage to care following initial screening, limiting the overall effectiveness of these programs in ensuring continued access to vision services. Policy measures, including provisions in the Affordable Care Act (ACA), have expanded vision coverage for pediatric populations; however, coverage for adult vision services is not uniformly mandated (17). Emerging strategies, such as teleophthalmology, have been used to connect patients in underserved communities with eye care providers, but challenges related to health literacy and patient engagement persist (18). Despite these initiatives, eye care in the United States remains fragmented across clinical, policy, and community-based domains (15). Overall, access to eye care differs by geographic location, health system capacity, and individual socioeconomic factors, rather than being consistently accessible across all populations (12).

This fragmentation must also be considered within the broader policy environment in which eye care services are funded and sustained. Federal initiatives, such as the Vision Health Initiative, support surveillance, public awareness, and program development. However, funding instability and shifting political priorities can limit the scope and sustainability of these efforts (15). For example, recent budget proposals included nearly $2 million in cuts to the Vision Health Initiative, raising concerns about the continuity of its surveillance systems and public health programs (19). At the same time, congressional action preserved funding for key research institutions such as the National Eye Institute, highlighting inconsistencies in federal prioritization (19). Such variation in federal policy support highlights the importance of the broader policy environment in efforts to address disparities in eye care access, particularly among low-income and disadvantaged populations.

Translating evidence of disparities into potential policy solutions remains an important consideration. To support this process, this paper offers a synthesis within a unified framework of findings on structural barriers, health care utilization, system capacity, and policy responses that are dispersed across disciplines and levels of analysis. It employs the Kingdon’s Multiple Streams Framework, which conceptualizes policy change as the alignment of problem recognition, available policy solutions, and political conditions (20). In this review, the framework is used as an interpretive lens for mapping the literature rather than as a formal policy process analysis. We offer a focused scoping review that maps literature identified through a deliberately high-specificity search strategy on disparities in eye care access in the United States, with particular attention to socioeconomic and insurance-related barriers, health system capacity, and policy and programmatic interventions to improve access. By synthesizing evidence across these domains, the review identifies key knowledge gaps to inform future research and policy efforts to reduce inequities in eye care. Integration of evidence across clinical, system, and policy domains highlights where breakdowns occur in translating evidence into policy action.

## Methods

### Study design

The scoping review was conducted in accordance with the Arksey and O’Malley (21) framework, as refined by Levac et al. (22), and the PRISMA-ScR checklist. An internal protocol specifying the search strategy, eligibility criteria, and screening procedures was developed prior to screening but was not prospectively registered. The primary research question addressed how income and related structural factors are associated with inequities in eye care access and what interventions have been implemented to mitigate these disparities. The findings are intended to inform policy and program development and to guide future research aimed at improving eye care access for underserved populations.

### Search strategy

A systematic search was conducted using PubMed, Scopus, CINAHL, and Google Scholar to identify relevant literature published between 2001 and 2025. The final database search was August 5, 2026 (Supplementary Table S2). PubMed was selected for its extensive coverage of biomedical and health services research, while Scopus provided broader interdisciplinary coverage of public health and policy literature. CINAHL was included to capture nursing, allied health, and health services literature that may not be indexed in PubMed or Scopus. Google Scholar was used as a supplementary search source to identify additional interdisciplinary and grey literature that may not be indexed in traditional bibliographic databases.

The search included studies published between 2001 and 2025 and used a string with combinations of keywords such as “eye care,” “income,” “health barriers,” and “vision health.” The search strategy was adapted for Google Scholar to account for differences in database functionality and its broader full-text indexing. Given the breadth and heterogeneity of literature addressing eye care access, socioeconomic disadvantage, and health policy, we used a deliberately high-specificity search strategy in which search terms were restricted to article titles as a pragmatic strategy to improve specificity and maintain a manageable and reproducible screening set. This approach prioritized studies explicitly focused on the review concepts but may have reduced sensitivity for studies in which relevant socioeconomic or access-related factors were reported only in abstracts or full text. The complete list of search terms is provided in Supplementary Table S1. Supplementary Table S2 summarizes the sources searched, dates of search, filters applied, fields searched, records retrieved, and records exported.

Because of the differences in the functionality and reproducibility of Google Scholar compared with traditional bibliographic databases, additional details regarding this search are provided. The Google Scholar search was conducted using Publish or Perish and was limited to publications from 2001 to 2025 (23). The search retrieved 647 results, all of which were screened for eligibility. Because results were retrieved through Publish or Perish rather than through manual review of Google Scholar result pages, a page-based stopping rule was not applied. The search was conducted while logged into Google. Backward reference searching and forward citation tracking were not systematically performed.

Records identified through the searches underwent title and abstract screening using predefined inclusion and exclusion criteria. Studies were considered eligible if they examined access to eye care among low-income populations in the United States or contributed relevant contextual evidence regarding eye care access and policy in urban or rural settings. Eligible study types included quantitative and qualitative empirical research, literature reviews, and relevant policy analyses.

### Inclusion criteria

- Geographic scope: Studies conducted in or addressing the United States.
- Income relevance: Studies examining income or poverty directly, income indicators of socioeconomic disadvantage, or barriers relevant to low-income or underserved populations.
- Study design: Quantitative or qualitative empirical studies, literature reviews, and policy analyses relevant to eye care access or health policy.
- Publication period: 2001–2025

### Exclusion criteria

- Studies examining broad health disparities without specific analysis of eye care or vision-related outcomes.
- Studies conducted outside the United States.

### Income-related eligibility and exposure measurement

Income was not required to be directly measured for study inclusion. Eligible studies either measured income or poverty directly, used an income-linked proxy such as Medicaid eligibility, or examined structural or policy barriers relevant to low-income populations. Direct measures included individual or household income, poverty ratios, and Federal Poverty Level categories. Income could be included as one of several covariates; a distinct income-stratified result was not required for inclusion. During data extraction, individual- and household-level measures were distinguished from ecological measures, such as area-level poverty or median household income. Supplementary Table S3 summarizes the income-related characteristics of the 17 sources identified through the database search. Accordingly, throughout this review, income-related refers broadly to evidence based on direct measures of income or poverty, income-linked indicators or proxies, and structural or policy conditions specifically affecting low-income populations; findings based directly on measured income or poverty are distinguished where conclusions concern income itself.

### Study selection

Study selection was conducted using the predefined inclusion and exclusion criteria described above. All records identified through the search were imported into Zotero, yielding 8,651 records. After removal of 1,314 duplicate records, including three records identified by automation tools, 7,337 records remained for title and abstract screening. One reviewer screened the titles and abstracts of all 7,337 records for eligibility. A formal calibration exercise or random duplicate-screening sample was not conducted at the title and abstract screening stage. A total of 7,191 records were excluded at this stage, leaving 146 articles for full-text review. One reviewer conducted the initial full-text assessment based on study type, study setting and population, methodological approach, and relevance to income-related disparities in eye care access. A second reviewer independently verified all full-text eligibility decisions. Disagreements were resolved through discussion and consensus. Of the 146 full-text articles assessed for eligibility, 129 were excluded based on predefined criteria, resulting in 17 publications identified through the database searches for inclusion in the review. Supplementary Table S4 summarizes the reasons for the exclusions at the full-text screening stage. The study selection process and number of records at each stage are presented in the PRISMA-ScR flow diagram (Figure 1). Two additional grey literature sources were identified outside the systematic search and purposively included to provide relevant policy and health-system context: H.R. 5,304, a federal legislative document, and a policy report. These sources provided context on federal health policy, funding, and health-system structures relevant to eye care access. Together with the 17 publications identified through the database searches, these sources resulted in a total of 19 sources included in the review. The rationale for inclusion and contribution of the two grey literature sources is presented in Supplementary Table S5.

**Figure 1:**
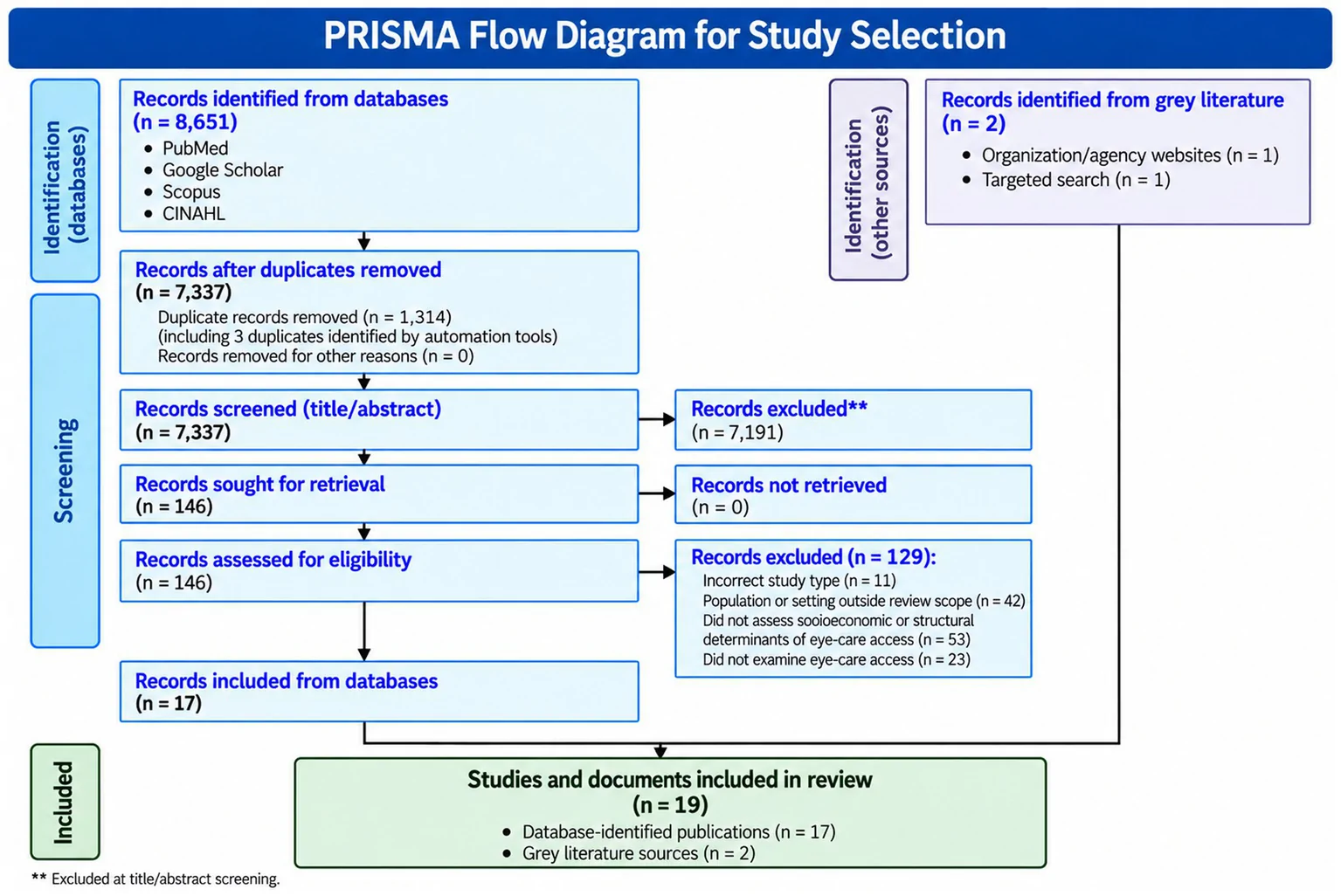
PRISMA flow diagram of the study selection process. The diagram summarizes the identification, screening, eligibility assessment, and inclusion of studies, including records removed before screening and reasons for full-text exclusion.

### Data charting

Data from the included sources were charted using a standardized data extraction form. Two reviewers independently extracted information on study characteristics, including author, publication year, journal, study design, study setting, study population, methodological approach, measures of income or socioeconomic disadvantage, and key findings. Any discrepancies in data extraction were resolved through discussion and consensus. Income-related characteristics were additionally documented to distinguish direct measures of income or poverty, income-linked proxies, and ecological measures of socioeconomic disadvantage. Supplementary Table S3 summarizes the income-related characteristics of the 17 sources identified through the database search.

### Critical appraisal

Critical appraisal of the 17 database-identified publications and the included grey literature policy report was conducted by a single reviewer using design-appropriate tools to assess methodological rigor (Supplementary Tables S7–S14). The appraisal was not independently duplicated or formally calibrated by a second reviewer. All appraisal tools were used exactly as published, without modification. The federal legislative document, H.R. 5,304, was not formally appraised because it is a legislative document rather than a research document. The methodological quality of each appraised source was evaluated using appraisal tools appropriate to its study design, including the Joanna Briggs Institute (JBI) Critical Appraisal Checklists, the Critical Appraisal Skills Programme (CASP) checklists, the Scale for the Assessment of Narrative Review Articles (SANRA), the Quality Assessment of Diagnostic Accuracy Studies-3 (QUADAS-3) tool, and the AACODS checklist for grey literature. Specifically, 7 studies were appraised using the JBI Analytical Cross-Sectional Studies Checklist (24), 3 using the CASP Descriptive Cross-Sectional Studies Checklist (25), 1 using the JBI Cohort Studies Checklist (26), 1 using the JBI Systematic Reviews of Qualitative Evidence Checklist (27), 1 using the JBI Evidence from Narrative, Opinion, and Policy Checklist (28), 3 using SANRA (29), 1 using the Quality Assessment of Diagnostic Accuracy Studies-3 (QUADAS-3) tool (30), and 1 grey literature report using the AACODS (Authority, Accuracy, Coverage, Objectivity, Date, and Significance) checklist (31).

Because the design-specific appraisal instruments assess different methodological domains and do not produce directly comparable global quality scores, appraisal findings were not converted into a standardized overall quality rating across studies. Instead, findings were synthesized descriptively by identifying recurring methodological strengths and limitations within and across study designs. Among quantitative studies, common strengths included clearly defined study populations, exposures, outcomes, and approaches to controlling for confounding. Common limitations included reliance on self-reported measures of vision status or health care utilization and the cross-sectional design of many studies, which limited causal inference. Narrative reviews and policy analyses were generally supported by relevant literature, although some did not report formal search strategies. The qualitative study demonstrated strengths across the methodological domains assessed, and the program evaluation and grey literature report met most applicable appraisal criteria. Given the heterogeneity of study designs, data sources, and appraisal instruments, these findings were considered descriptively rather than combined into an overall methodological quality category. Critical-appraisal findings were used to contextualize interpretation of the evidence rather than as criteria for study exclusion, weighting, or Kingdon stream assignment. No study was excluded on the basis of methodological appraisal. The narrative synthesis considered identified methodological limitations when interpreting the strength and consistency of findings across the included literature.

### Application of Kingdon’s multiple streams framework

Following study selection and data extraction, Kingdon’s Multiple Streams Framework was applied to support the interpretive synthesis of findings. Operational definitions were developed for the problem, policy, and politics streams. The problem stream included evidence of income-related inequities, unmet needs, barriers, disparities, disease burden, and failures in access to eye care. The policy stream included evidence describing proposed or implemented approaches to improving access, including financing mechanisms, screening programs, teleophthalmology, and other interventions. The politics stream included evidence related to political priorities, legislation, Medicaid policy, funding decisions, stakeholder support, the implementation environment, and the political feasibility of efforts to improve eye care access. Each included source was coded according to the stream or streams to which its findings contributed. Sources could be assigned to more than one stream when the reported evidence met the operational definitions for multiple streams.

Kingdon’s multiple stream coding was initially conducted by one reviewer using the prespecified operational definitions. A second reviewer independently verified the stream assignments for all included sources. Disagreements were resolved through discussion and consensus. The stream coding did not influence study eligibility or the initial extraction of findings. The coding of included sources across the three streams is presented in Supplementary Table S15. The framework was subsequently used to examine areas of convergence across the problem, policy, and politics streams. Convergence across the streams was interpreted as a potential policy opportunity rather than empirical evidence that a policy window had opened. Findings reported by the included sources were distinguished from policy implications derived through application of the framework; accordingly, proposed policy opportunities represent author interpretations of the evidence synthesis rather than findings directly established by the included studies. In addition, because the literature-identification strategy did not include a systematic search of government, legislative, advocacy, or other policy-focused grey literature, the relative representation of the three streams was interpreted only as a characteristic of the included evidence base and not as an indication of their prominence or alignment within the broader U.S. policy environment.

## Results

The 19 sources identified through the prespecified search and supplementary grey literature inclusion addressed how income and related structural factors are associated with inequities in eye care access. The primary objective was to characterize income-related barriers to eye care and identify interventions designed to improve access among low-income populations. To contextualize the translation of these barriers and interventions into policy action, findings were interpreted using the Kingdon’s Multiple Streams Framework, which conceptualizes policy change as the convergence of problem recognition, policy solutions, and political conditions (Figure 2) (20).

**Figure 2:**
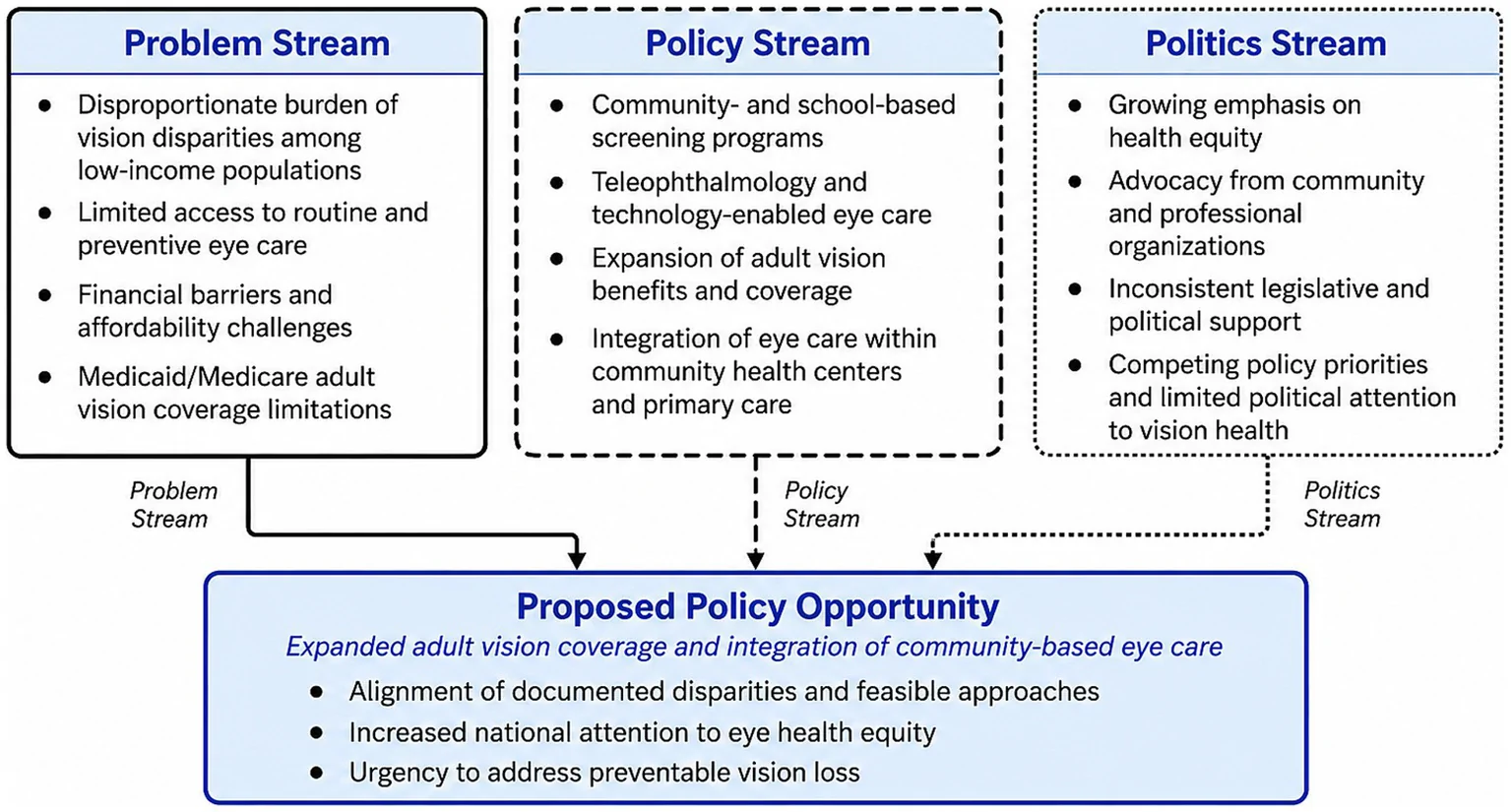
Conceptual synthesis of findings using Kingdon’s Multiple Streams Framework. The figure organizes findings from the included evidence across the problem, policy, and politics streams and illustrates author-interpreted potential policy opportunities. It should not be interpreted as empirical evidence that the three streams are currently converging or that a policy window is open.

### Problem recognition stream: income-related disparities in vision health

Across the literature, disparities affecting low-income populations were documented through several forms of evidence, including direct measures of income or poverty, income-linked indicators such as insurance or Medicaid status, and broader structural barriers affecting economically disadvantaged populations. Studies directly measuring income or poverty generally identified associations between lower income and poorer vision outcomes or reduced access to eye care, while studies using income-linked proxies and structural measures identified additional barriers related to insurance coverage, system capacity, and access to services (1–7, 11–14). Evidence from national surveys and studies of community-based programs indicates that disparities are observed among individuals with lower income across multiple domains, including a higher prevalence of visual impairment, reduced access to preventive eye care, and greater reliance on delayed or acute care services (1–6, 13, 14). Across several included studies, lower-income, uninsured, and publicly insured populations were less likely to access routine outpatient care and more likely to report delayed treatment, greater reliance on emergency services, or more advanced disease at presentation (1, 2, 5, 6, 13, 14). These disparities are seen across the life course, affecting both adult and pediatric populations, and are evident in both clinical outcomes and patterns of care utilization (1–4, 13, 14). These disparities reflect cumulative barriers associated with differences in how and when individuals access preventive and routine eye care services (1, 2, 5, 6, 13, 14). Taken together, studies directly measuring income suggest associations between lower income, health outcomes, and health care utilization, while the broader evidence demonstrates interconnected financial, insurance, and structural barriers to eye care access (1–7, 11–14). Within Kingdon’s Multiple Streams Framework, these findings provide evidence for the problem stream, documenting persistent income-related inequities in both health outcomes and access to care.

### Policy stream: existing interventions and programmatic responses

The literature identifies a range of interventions intended to improve access to eye care for low-income populations, spanning community-based programs, clinical service delivery models, and focused initiatives intended to reduce financial and structural barriers to care (15–18). Collectively, these sources describe multiple approaches that have been implemented to improve eye care access across community and clinical settings (15–18).

Community-based vision screening and outreach initiatives have been associated with increased screening and identification of unmet vision needs in underserved populations. Interventions described in the literature target multiple age groups, including pediatric and older adult populations (15, 16).

Across the included evidence, some interventions were associated with improved access or service utilization in specific populations and settings (16, 18). From the perspective of Kingdon’s Multiple Streams Framework, these findings suggest that although potential policy solutions have been identified, they have not been consistently integrated into broader state and federal health care systems. The policy stream contains multiple approaches to improving eye care access; however, many interventions remain dependent on community organizations, temporary funding, or individual health systems rather than broader policy adoption. Community health centers, for example, require sustained federal investment and adequate Medicaid reimbursement to expand services (12). Together, these findings highlight long-term funding mechanisms, implementation, and broader evaluation of promising strategies across health systems as important areas for further consideration.

### Politics stream: policy context and structural constraints

The literature illustrates how the broader political and health system environment is associated with access to eye care, particularly through variations in insurance coverage, system prioritization, and funding allocation. Included sources indicate recognition of eye care disparities at the federal and public health levels, although the evidence reviewed does not demonstrate consistent translation into comprehensive, coordinated policy action (15, 19).

Insurance policy design, including state-level variation in Medicaid coverage and the limited inclusion of routine eye care within public insurance programs, is associated with differences in access to services (5, 6, 17). These findings suggest that variation in Medicaid coverage is associated with geographic variation in eye care access and the implementation of eye care services across states. In addition, the prioritization of eye care within broader health systems remains inconsistent, and service availability is uneven across settings (15, 19).

Community-level advocacy and health system engagement, including efforts led by local health centers and organizations, are among the approaches described for addressing gaps in access to care (7, 12, 15, 16). The prominent role of community health centers and local organizations highlights the importance of community-based service delivery. However, their continued ability to expand and sustain access remains closely tied to federal investment and Medicaid policy (12). Within the politics stream, these findings suggest that recognizing eye care disparities is only one component of policy change. Funding support and policy prioritization may therefore be important considerations.

### Proposed policy opportunity

Application of the coding framework indicated that included sources often contributed evidence to more than one stream (Supplementary Table S15). Of the 19 included sources, 17 (89%) contributed to the problem stream, 16 (84%) to the policy stream, and 9 (47%) to the politics stream. Seven sources (37%) contributed to all three streams, nine (47%) to two streams, and three (16%) to a single stream. Evidence within the included sources was most frequently mapped to the problem and policy streams, reflecting greater representation of evidence concerning disparities and proposed or implemented approaches within our search. Within the literature identified through this review, comparatively fewer sources contributed evidence to the politics stream. Given that the search strategy primarily targeted peer-reviewed literature and did not systematically search government, legislative, advocacy, or other policy-focused grey literature, this pattern should be interpreted as the distribution of evidence within the included sources rather than as evidence of limited political activity or engagement. Taken together, the included evidence identifies disparities in access and describes several proposed or implemented approaches to addressing them. Application of Kingdon’s framework was then used to interpret the potential policy implications of these findings. This interpretive synthesis suggests expanded adult vision coverage and greater integration of community-based eye care as potential policy opportunities. These opportunities represent author-derived implications of the evidence synthesis rather than findings directly evaluated by the included studies, and the available evidence was not considered sufficient to establish that a policy window is currently open. Instead, this alignment was interpreted as a proposed policy opportunity for expanded adult vision coverage and integration of community-based eye care.

## Discussion

### Summary of main findings

Across the heterogeneous evidence identified in this review, multiple studies reported limited access to eye care among low-income populations (1–6, 13, 14). Across studies, disparities were associated with limited provider availability within safety-net systems (7, 12), gaps in insurance coverage (5, 6, 17), and low health literacy (9, 10, 18). Informational barriers and low levels of health literacy are also associated with disparities in eye care utilization and follow-up adherence (9, 10, 18). These barriers occur alongside uneven availability of community-based initiatives in underserved areas (7, 10, 15).

Importantly, the evidence supporting these findings varied in how income-related disadvantage was operationalized. Some studies directly measured individual or household income or poverty, whereas others relied on income-linked proxies, such as Medicaid status, or examined structural conditions disproportionately affecting low-income populations. Conclusions specifically concerning income are therefore based primarily on studies that directly measured income or poverty, while the broader synthesis reflects the interconnected financial, insurance, and structural conditions associated with access among low-income populations. The strength and interpretation of these findings also vary by study design; observational studies primarily identify associations, whereas program evaluations and intervention-focused studies provide evidence regarding implementation or changes in access within specific settings and should not be interpreted as establishing population-level effectiveness.

Disparities in eye care and vision health affect both individual and population health (1, 3, 4, 13). Previous literature has associated visual impairment with reduced quality of life, depression, injuries, and loss of productivity (1, 15). In pediatric populations, disparities in access to eye care may additionally affect educational experiences and healthy child development (4, 15). At the population level, inadequate access to preventive eye care may contribute to preventable health care utilization and broader economic burden associated with untreated vision impairment (13, 15).

Despite longstanding recognition of disparities in eye care access, these inequities remain evident in the United States. The findings suggest that recognition of the problem alone has not been accompanied by widespread improvements in access to care (7, 12, 15, 16, 18). Although multiple interventions have been associated with improved access, most remain localized and depend on temporary funding or community organizations rather than being integrated into the broader health care system (7, 12, 15–18). Community-based screening programs and teleophthalmology initiatives illustrate this challenge through their association with improved access in underserved settings while remaining largely resource-dependent and not yet broadly implemented (7, 12, 15, 16, 18). Strengthening integration between primary care providers and eye care specialists may improve early detection and care coordination for low-income populations (15, 18). Differences in Medicaid vision benefits and reimbursement policies are also associated with variation in access across states (5, 7, 12, 17). Based on these findings, we interpret expanded adult vision benefits, provider reimbursement, and reduced variation in coverage across states as potential policy considerations. These implications extend from, but were not directly evaluated by, the included evidence and therefore require further policy and implementation evaluation. In addition, provider shortages, transportation barriers, and limited health literacy continue to be associated with access challenges for low-income populations (7, 9, 10, 12, 18). Through the lens of Kingdon’s Multiple Streams Framework, the included evidence provides representation of the problem and policy streams. Politics-stream evidence was less frequently represented within the review; however, given the literature-identification strategy, this should not be interpreted as evidence of limited political activity or prioritization. The evidence included also indicates that many identified interventions remain localized or have not been widely integrated into broader health care systems. Taken together, the synthesis highlights potential gaps in alignment among financing, stakeholder priorities, and policy support. Greater convergence among these streams may increase opportunities for policy action on comprehensive adult vision coverage (19, 20).

As contextual evidence external to the prespecified review evidence base, international experience provides an example of the potential benefits of integrating eye care into publicly funded health systems. Rwanda provides a relevant example because it has integrated eye care into a national community-based health insurance scheme alongside a nationwide primary eye care program, illustrating a model in which financing and service delivery are coordinated within a national eye care strategy. This coordinated national strategy was associated with expanded access to affordable eye care, strengthened referral pathways, and increased demand for secondary and tertiary eye care services following improvements in primary eye care access (32). However, Rwanda’s experience should be viewed as an illustrative example rather than a directly transferable model, given differences in health-system organization, financing, and governance between Rwanda and the U.S. health care system.

Although this review focused on income-related disparities, relatively few studies examined income as an isolated factor associated with eye care access. Instead, income was frequently considered alongside other social determinants of health, including insurance status, educational attainment, geographic access, transportation, and health literacy. This reflects the interconnected nature of socioeconomic disadvantages and suggests that inequities in eye care are best understood as being associated with multiple interconnected structural factors rather than income alone.

### Limitations

This review has several limitations that should be considered when interpreting the findings. First, although PubMed, Scopus, Google Scholar, and CINAHL provided broad coverage of the relevant literature, studies indexed exclusively in other databases may not have been captured. Two sources of grey literature were purposively included to provide additional policy and health-system context. However, unpublished studies and reports not identified through the search strategy may still have been missed. Second, the included evidence varied in study design, population, outcomes, and measures. This heterogeneity precludes quantitative synthesis. Findings were therefore synthesized narratively with an emphasis on patterns rather than the magnitude of effects. Third, the review was limited to studies conducted in the United States in accordance with the study objective. This may limit the generalizability of the findings to other health systems and policy contexts. Fourth, the search strategy deliberately prioritized specificity over sensitivity by restricting search terms to article titles, and backward-reference searching and forward-citation tracking were not systematically conducted. These decisions may have reduced search sensitivity and resulted in relevant studies being missed, particularly studies in which income, socioeconomic disadvantage, insurance, or access barriers were reported primarily in the abstract or full text. The findings should therefore be interpreted as a mapping of literature identified through the prespecified search strategy rather than an exhaustive representation of all potentially relevant evidence. Fifth, the literature-identification strategy may also have influenced the application of Kingdon’s Multiple Streams Framework. Government documents, legislative materials, advocacy reports, state policy sources, and other grey literature were not systematically searched and may contain politics-stream evidence not captured in this review. The comparatively smaller representation of the politics stream should therefore be interpreted as a characteristic of the included evidence base rather than evidence of limited political activity or engagement. Finally, initial title and abstract screening was conducted by a single reviewer without formal calibration or duplicate screening, which may have introduced selection bias and increased the possibility that relevant studies were inadvertently excluded. This limitation was partially mitigated at subsequent stages through verification of all full-text eligibility decisions by a second reviewer and independent data extraction by two reviewers. Critical appraisal was also conducted by a single reviewer without independent verification, introducing the possibility of reviewer judgment in appraisal decisions; however, appraisal findings were used to contextualize interpretation and were not used for study exclusion. The findings of this work should be interpreted considering the relatively small and heterogeneous evidence base, the limited number of policy-focused sources, restrictions inherent in the databases and search methods used, and methodological weaknesses identified across some included sources. Although the literature identifies several promising approaches to improving access, the available evidence does not establish their scalability, long-term sustainability, cost-effectiveness, or population-level effectiveness.

### Future directions

The following considerations draw on contextual literature outside the prespecified evidence base and are presented to identify potential directions for future research rather than as findings of the scoping review. Future implementation research should evaluate strategies for delivering community- and school-based vision screening programs, expanding teleoptometry and teleophthalmology services to rural and underserved populations, and translating clinical evidence into sustainable health system and policy change (33).

Expanding Medicaid coverage to include comprehensive vision services and eyeglass distribution would require policy changes, additional public investment, and greater standardization of benefits across states that currently treat routine eye care as optional. Existing pediatric vision coverage provides one policy model that could inform consideration of coverage for a broader range of age groups. However, major challenges to such changes include provider availability, cost-shifting, and consistent administrative control.

Beyond the policy approaches identified in the review corpus, emerging technologies may warrant future evaluation as potential strategies for addressing access and service-capacity barriers affecting underserved populations. Contextual literature external to the review evidence base suggests that artificial intelligence-based screening and teleophthalmology may offer opportunities to expand screening capacity and access in settings with limited eye care services. Because widespread policy change may take years, strategies that can address more immediate gaps in access also warrant consideration. New and developing technologies may help expand screening and service capacity across socioeconomic groups. Artificial intelligence-based software is increasingly being used in vision screening to detect a range of eye diseases and conditions. AI software systems have demonstrated promising performance in detecting diabetic retinopathy and age-related macular degeneration. Examples of AI systems that have been used in clinical settings include EyeArt, Thirona Retina, IDx-DR, and IGrading M (34). These systems have the potential to expand screening capacity in communities with limited access to eye care providers by enabling appropriately trained non-eye care professionals to perform initial screening and referral. Teleophthalmology is an emerging approach that may improve access for individuals who face transportation-related barriers to medical facilities. However, large-scale implementation may be limited by equipment costs, unreliable electricity and internet access, and patient acceptance in some settings (35). Despite these challenges, teleophthalmology remains a promising approach for expanding access to individualized eye care.

## Conclusion

Disparities in access to eye care among low-income populations in the United States remain evident and are associated with a combination of structural, financial, and informational barriers. Limited provider availability, gaps in insurance coverage, and uneven access to community-based resources are associated with reduced utilization of preventive services and poorer vision health outcomes.

Addressing these disparities will likely require coordinated, system-level approaches that move beyond localized or short-term interventions. The findings suggest potential policy opportunities related to greater alignment between identified needs, available interventions, and policy priorities, alongside stronger integration of eye care within broader health systems. Policy implications emerging from this synthesis include consideration of expanded Medicaid and other public insurance coverage for routine vision services and greater integration of vision screening and preventive eye care into primary care settings. These considerations reflect gaps and promising approaches identified across the literature; however, the available evidence does not establish their cost-effectiveness, scalability, long-term sustainability, or population-level effectiveness if implemented nationally.

## Data Availability

The original contributions presented in the study are included in the article/Supplementary material, further inquiries can be directed to the corresponding author/s.
