# Supplementary material for: Blinded by inequity: a scoping review of income-related and structural disparities in eye care in the United States
Source: Front Public Health. 2026 Oct 19;14:1885854. doi: 10.3389/fpubh.2026.1885854 (PMC13724332; doi:10.3389/fpubh.2026.1885854)
Supplement: Supplementary file 1 [file Supplementary_file_1.docx]

**Supplementary Material**

| **Supplementary Table S1. List of Search Terms** | |
| --- | --- |
| **Database** | **Search Terms** |
| PubMed | “eye care" OR "eye care services" OR "vision impairment" OR ophthalmolog* OR optometr* OR "vision health" OR "ophthalmic" OR "eye health" OR "essential health benefits" OR teleophthalm* OR teleretinal*) AND (access* OR disparit* OR Medicaid OR Medicare OR insurance OR polic* OR income* OR health equity OR underserved populations OR socioeconomic barriers |
| Google Scholar | “eye care" OR "eye health" OR ophthalmology OR optometry) (access OR disparities OR income OR socioeconomic OR insurance OR Medicaid OR Medicare) |
| Scopus | “eye care" OR "eye care services" OR "vision services" OR "vision impairment" OR ophthalmolog* OR optometr* OR "vision health" OR "ophthalmic" OR "eye health" OR "essential health benefits" OR teleophthalm* OR teleretinal* AND access* OR disparit* OR Medicaid OR Medicare OR insurance OR polic* OR income* OR health equity OR “underserved populations” OR “socioeconomic barriers” |
| CINAHL | "eye care" OR "eye care services" OR "vision services" OR "vision impairment" OR ophthalmolog* OR optometr* OR "vision health" OR "ophthalmic" OR "eye health" OR "essential health benefits" OR teleophthalm* OR teleretinal* AND access* OR disparit* OR Medicaid OR Medicare OR insurance OR polic* OR income* OR health equity OR “underserved populations” OR “socioeconomic barriers” |

| **Supplementary Table S2. Summary of Databases Searched, Search Dates, Filters Applied, Fields Searched, Records Retrieved, and Records Exported to Zotero** | | | | | | |
| --- | --- | --- | --- | --- | --- | --- |
| **Database** | **Date Searched** | **Filters/Limits** | **Controlled Vocabulary Used?** | **Fields Searched** | **Records Retrieved** | **Records Exported** |
| PubMed | 10/15/2025 | Studies Published from 2001 to 2025 | No | Title/Abstract | 7,234 | 7,234 |
| Google Scholar | 10/15/2025 | Studies Published from 2001 to 2025 | No | Title | 647 | 647 |
| Scopus | 10/15/2025 | Studies Published from 2001 to 2025 | No | Title, Abstract, and Keywords | 513 | 513 |
| CINAHL | 08/05/2026 | Studies Published from 2001 to 2025 | No | Title | 257 | 257 |

| **Supplementary Table S3. Income-Related Characteristics of Sources Identified Through the Database Search** | | | | | | |
| --- | --- | --- | --- | --- | --- | --- |
| **Title** | **Author** | **Income-Measure** | **Level of Income Measure** | **Definition of Low-Income Group** | **Analytical Role of Income** | **Direction of Association** |
| Socioeconomic Disparity in Use of Eye Care Services Among US Adults With Age-Related Eye Diseases: National Health Interview Survey, 2002 and 2008 | Zhang et al. | Poverty-income ratio (PIR) | Individual and household | Lowest income category: PIR <1.50; | Main independent variable. Analyzed categorically in multivariable logistic regression | Participants with PIR <1.50 were significantly less likely than those with PIR ≥5.00 to visit an eye-care provider |
| Eye Care Among US Adults at High Risk for Vision Loss in the United States in 2002 and 2017 | Saydah et al. | Income-to-poverty ratio (IPR) | Individual and household | Poor: IPR <1.0, near poor: IPR 1.0–<2.0, not poor: IPR ≥2.0 | Covariate/independent predictor in multivariable logistic regression | Lower income was associated with lower eye-care utilization and greater inability to afford eyeglasses |
| Disparities in Vision Impairment Among Adults in the United States | Wilson et al. | Annual household income | Individual and household | Lowest income category: <$25,000/year | Covariate/independent predictor in multivariable logistic regression | Lower household income associated with greater odds of vision impairment |
| Disparities in Vision Health and Eye Care | Elam et al. | Variation (SES across cited studies: family income, poverty level, and area-level poverty) | Variation (household and geographic location) | Definitions varied across cited studies | Income examined as a social determinant of vision health and eye-care disparities in a narrative synthesis | Lower income consistently associated with greater vision impairment and reduced access |
| Most State Medicaid Programs Cover Routine Eye Exams for Adults, But Coverage of Other Routine Vision Services Varies | Lipton et al. | Medicaid enrollment/eligibility as a proxy for low income | Program eligibility | Medicaid-enrolled adults | Population eligibility criterion (Medicaid enrollment) | Substantial gaps in vision coverage were identified among Medicaid enrollees |
| Comparison of Access to Eye Care Appointments Between Patients With Medicaid and Those With Private Health Care Insurance | Lee et al. | Medicaid insurance status and location median household income | Geographic location | Medicaid vs private insurance, lowest geographic household income category: $30,001–$50,000 | Medicaid status was the primary exposure and regional median household income was a covariate in multivariable regression | Medicaid associated with lower appointment access |
| Assessing the Need for On-Site Eye Care Professionals in Community Health Centers | Shin et al. | Federal Poverty Level (FPL) | Individual income, reported in aggregate | ≤100% FPL | Population characteristic | Not directly assessed, community health centers serving predominantly low-income populations had substantial limitations in eye care capacity |
| Sociodemographic Factors Associated With Emergent Eye-Related Emergency Department Visits: A Multicenter Analysis | Quintero et al. | Insurance status (Medicaid and uninsured status as proxies for socioeconomic disadvantage) | Individual level proxy | Medicaid enrolled and uninsured patients, no direct income threshold defined | Insurance status examined as an independent predictor of emergent versus nonemergent eye related ED visits | Uninsured patients were more likely to have emergent diagnoses than patients with private insurance, Medicare, or Medicaid |
| Public Attitudes About Eye and Vision Health | Scott et al. | No direct income measure reported | Not applicable | Lower income respondents included through presurvey stratification; no threshold reported | Sampling consideration only | Not assessed |
| The Importance of Health Literacy in Addressing Eye Health and Eye Care Disparities | Capo et al. | Multiple income and socioeconomic measures across included literature | Varied across cited studies | No single definition; varied across cited studies | Socioeconomic determinant examined within narrative synthesis | Lower income associated with lower eye health awareness and reduced access to health information and digital resources |
| Disparities in delivery of ophthalmic care; An exploration of public Medicare data | Lee et al. | No income measure reported | No income measure reported | No low income group defined; study population consisted of Medicare beneficiaries | Income not analyzed; geographic access was the primary exposure | No income association assessed; greater distance to providers was associated with lower cataract care utilization |
| Disparities in Access to Medical Care for Individuals with Vision Impairment | Spencer et al. | Total annual income, adjusted to 2004 dollars | Individual | Lowest income quartile: <$9,707; income categorized into four quartiles | Independent covariate in multivariable logistic regression | Higher income was associated with greater likelihood of having a usual source of care and lower likelihood of several access barriers |
| Demographic and Socioeconomic Differences in Outpatient Ophthalmology Utilization in the United States | Rasendran et al. | Household income relative to the 2015 Federal Poverty Level (FPL) | Individual/household | Poor: <100% FPL, near poor: 100–124% FPL, low income: 125–199% FPL | Independent predictor in multivariable logistic regression | Lower income associated with lower outpatient ophthalmology utilization |
| Making Eye Health a Population Health Imperative: Vision for Tomorrow (2016) | National Academies of Sciences, Engineering, and Medicine | Socioeconomic status, poverty, and income-based public insurance eligibility | Individual/household and geographic/population-level | Varied across cited evidence, included low socioeconomic status, poverty, and Medicaid/CHIP income-eligible populations | Social/economic determinant synthesized from existing evidence, not independently analyzed by report | Lower socioeconomic status and poverty associated with greater vision disparities and barriers to eye care access |
| Outcomes of a Vision Screening Program for Underserved Populations in the United States | Friedman et al. | Not directly measured, underserved socioeconomic status characterized through recruitment from FQHC | No individual or area-level income variable measured | No explicit income threshold, study population was defined as underserved and recruited from a free clinic and federally qualified health center | Not analyzed | No association between income and study outcomes was tested |
| Essential Health Benefits and the Affordable Care Act: Law and Process | Bagley et al. | Not measured | Not measured | No low income group defined | Not analyzed; source contributed policy evidence on insurance benefit design | No income association assessed |
| Understanding the Knowledge Gap Experienced by U.S. Safety Net Patients  in Teleretinal Screening | George et al. | Monthly individual income | Individual | <$1,000/month; 62% of participants earned below the 2012 poverty level of $11,170/year | Participant characteristic used to characterize socioeconomic barriers in the qualitative study population | Low income and financial pressures contributed to barriers to accessing care and following recommended treatment plans |

| **Supplementary Table S4. Summary of Reasons for Exclusion at the Full-Text Screening Stage** | | | |
| --- | --- | --- | --- |
| **Title** | **Author** | **Year** | **Reason for Exclusion** |
| A narrative review of neuro-ophthalmologic disease in African Americans and Hispanics with multiple sclerosis | Tardo et al. | 2023 | Wrong study focus. Focuses on neuro-ophthalmologic outcomes in multiple sclerosis rather than disparities in access to eye care among low-income populations. |
| A narrative review of telemedicine and its adoption across specialties | Garcia et al. | 2024 | Not specific to eye care disparities |
| A Need for More Equity in Prevention of Blindness | Blanchet et al. | 2015 | Not specific to the U.S. population |
| A Prospective Analysis of the Simplified Student Sight Savers Program on Open-Angle Glaucoma Cost Burden in Underserved Communities | Bradshaw et al. | 2022 | Insufficient relevance to income-related or structural disparities in eye-care access. |
| A Randomized Controlled Trial Assessing the Effectiveness of Strategies Delivering Low Vision Rehabilitation: Design and Baseline Characteristics of Study Participants | Christy et al. | 2010 | Study conducted outside the United States. |
| A Randomized, Controlled Trial to Test the Effectiveness of a Glaucoma Patient Navigator to Improve Appointment Adherence | Hark et al. | 2016 | Insufficient relevance to income-related or structural disparities in eye-care access. |
| A Review on the Incidence and Related Risk Factors of Retinopathy of Prematurity Across Various Countries | Nair et al. | 2022 | Not specific to the United States or U.S. low-income populations |
| A Scoping Review of Patients’ Barriers to Eye Care for Glaucoma and Keratitis | Hicks et al. | 2023 | Insufficient relevance to income-related or structural disparities in U.S. eye-care access. |
| A Comparison of Black and Non-Black Patients in the Presentation and Treatment of Keratoconus | Christensen et al. | 2024 | Insufficient relevance to income-related or structural disparities in eye-care access. |
| A Simple Method for Estimating the Economic Cost of Productivity Loss Due to Blindness and Moderate to Severe Visual Impairment | Eckert et al. | 2015 | Insufficient relevance to income-related or structural disparities in U.S. eye-care access. |
| A Singular Focus on Insurance Coverage Will Not Lead to Eye Health Equity | Higginbotham | 2017 | Ineligible study design |
| A Study Establishing Sensitivity and Accuracy of Smartphone Photography in Ophthalmologic Community Outreach Programs: Review of a Smart Eye Camera | Andhare et al. | 2023 | Study conducted outside the United States. |
| A Survey of Public Eye-Care Behavior and Myopia Education | Liu et al. | 2025 | Study conducted outside the United States. |
| A Systematic Review of Current Teleophthalmology Services in New Zealand Compared to the Four Comparable Countries of the United Kingdom, Australia, United States of America (USA) and Canada | Walsh et al. | 2021 | Insufficient relevance to income-related or structural disparities in U.S. eye-care access. |
| A Systematic Review of Economic Evaluations Conducted for Interventions to Screen, Treat, and Manage Retinopathy of Prematurity (ROP) in the United States, United Kingdom, and Canada | Smith et al. | 2023 | Insufficient relevance to income-related or structural disparities in U.S. eye-care access. |
| A Vision for Universal Eye Health | The Lancet | 2019 | Outside the United States |
| A Compressive Assessment of Patient Satisfaction with Ophthalmology Clinic Services: A Study at a Tertiary Care Academic Institution Amidst and After the COVID-19 Pandemic | Asodariya et al. | 2024 | Outside the United States |
| Addressing Health Disparities Through Promoting Equity for Individuals with Intellectual Disability | Ouellette-Kuntz et al. | 2005 | Not specific to eye care disparities |
| A Cross-Sectional Retrospective Analysis of the Racial and Geographic Variations in Cataract Surgery | Shahbazi et al. | 2015 | Insufficient relevance to income-related or structural disparities in eye-care access. |
| Adopting a Comprehensive Approach to Ensure the Delivery of Gender-Specific Eye Healthcare Services in Low- and Middle-Income Nations | Shrivastava et al. | 2025 | Outside the United States. |
| Advancing Equity in Global Ophthalmology | Olivieri et al. | 2021 | Insufficient relevance to income-related or structural disparities in U.S. eye-care access. |
| Advancing Eye Health Within Universal Health Coverage: The Role of Programmatic Data and Implementation Research | Schmidt et al. | 2022 | Outside the United States |
| Acceptance and Perception of Artificial Intelligence Usability in Eye Care (APPRAISE) for Ophthalmologists: A Multinational Perspective | Gunasekeran et al. | 2022 | Insufficient relevance to income-related or structural disparities in U.S. eye-care access. |
| Advancing Inclusive Research (AIR) Site Alliance: Facilitating the Inclusion of Historically Underrepresented People in Oncology and Ophthalmology Clinical Research | Vidal et al. | 2024 | Wrong area of impact |
| Association Between Inequalities in Human Resources for Health and All Cause and Cause Specific Mortality in 172 Countries and Territories, 1990–2019: Observational Study | Yan et al. | 2023 | Not specific to eye care |
| Autonomous Artificial Intelligence for Diabetic Eye Disease Testing Improves Access and Equity in the Pediatric and Adult Populations: The Johns Hopkins Medicine Experience | Liu et al. | 2025 | Insufficient relevance to income-related or structural disparities in eye-care access. |
| Autonomous AI for Diabetic Eye Disease at Primary Care Improves Ophthalmic Access for At-Risk Patients | Liu et al. | 2024 | Insufficient relevance to income-related or structural disparities in eye-care access. |
| Barriers to Receiving Follow-Up Eye Care and Detection of Non-Glaucomatous Ocular Pathology in the Philadelphia Glaucoma Detection and Treatment Project | Zheng et al. | 2016 | Insufficient relevance to income-related or structural disparities in eye-care access. |
| Beyond Accessibility in Exploring Access to Eye Care to Achieve Vision Health Equity | Hicks et al. | 2023 | Commentary rather than an eligible research or review study |
| The Burden of Age-Related Macular Degeneration: Results of a Cohort Study in Two French Referral Centres | Bonastre et al. | 2003 | Outside the United States |
| Changes in Payer Mix Associated With Private Equity Acquisition of Ophthalmology Practices | Connolly et al. | 2025 | Insufficient relevance to income-related or structural disparities in eye-care access. |
| Changes in Utilisation of Optometry Services With a Non-Targeted Publicly-Funded Health Care Voucher System | Sum et al. | 2023 | Outside the United States |
| Community Ophthalmology – Can Excellence, Efficiency, and Equity Co-exist? | Honavar et al. | 2020 | Outside the United States |
| The Challenges in Improving Outcome of Cataract Surgery in Low and Middle Income Countries | Lindfield et al. | 2012 | Outside the United States |
| Community-Based Interventions to Detect Visual Impairment in Community-Dwelling Older Adults Aged ≥75 Years: A Systematic Review | Jensen et al. | 2025 | Population not specific to the United States |
| Comparative Analysis of LLMs in Dry Eye Syndrome Healthcare Information | Wu et al. | 2025 | Wrong area of impact |
| Considerations in Building a School-Based Vision Program | Ambrosino et al. | 2024 | Ineligible study design |
| Coronavirus Disease 2019–Related Health Disparities in Ophthalmology with a Retrospective Analysis at a Large Academic Public Hospital | Chung et al. | 2022 | Wrong area of impact |
| Cost-effectiveness of AI for pediatric diabetic eye exams from a health system perspective | Ahmed et al. | 2025 | Wrong population |
| Data Sources for Evaluating Health Disparities in Ophthalmology: Where We Are and Where We Need to Go | Baxter et al. | 2022 | Wrong area of impact |
| Diabetic Eye Care: Need and Opportunity for Systematic Change to Improve Access | Hanna et al. | 2016 | Outside the United States |
| The Evaluation of Screening Policies for Diabetic Retinopathy Using Simulation | Davies et al. | 2002 | Outside the United States |
| Disparities Between Teleretinal Imaging Findings and Patient-Reported Diabetic Retinopathy Status and Follow-up Eye Care Interval: A 10-Year Prospective Study | Silva et al. | 2024 | Insufficient relevance to income-related or structural disparities in eye-care access. |
| Addressing Health Disparities in Pediatric Eye Care for School-Age Children: A Call to Action | Antonio-Aguirre et al. | 2023 | Ineligible study design |
| Disparities in Vision Screening in Primary Care for Young Children With Autism Spectrum Disorder | Hoover et al. | 2023 | Insufficient relevance to income-related or structural disparities in eye-care access. |
| Does a Non-Targeted Publicly-Funded Health Care Voucher System for the Elderly Improve Access to Optometry Services? | Sum et al. | 2020 | Outside the United States |
| Economic Evaluations of Eye Care Services for Indigenous Populations in High-Income Countries: A Scoping Review | Nejatian et al. | 2024 | Geographic scope was not specific to the United States. |
| Everyday Challenges for Individuals Aging With Vision Impairment: Technology Implications | Remillard et al. | 2024 | Wrong area of impact |
| Exploration of Disparities in Regions and Specialized Fields of Day Surgery System | Gavurova et al. | 2020 | Outside the United States |
| Exploring Factors behind Patient Nonadherence to Intravitreal Anti-Vascular Endothelial Growth Factor Injections in Macular Diseases | Naaman et al. | 2025 | Outside the United States |
| Exploring Patient Involvement in Treatment Decision-Making for Diabetic Retinopathy: A COM-B Model-Based Cross-Sectional Survey | Xu et al. | 2025 | Outside the United States |
| Eye Care Delivery Models to Improve Access to Eye Care for Indigenous Peoples in High-Income Countries: A Scoping Review | Burn et al. | 2021 | Geographic scope not specific to the United States |
| Eye Care Disparities and Health Related Consequences in Elderly Patients with Age-Related Eye Disease | Umfress et al. | 2016 | Ineligible study design |
| Eye Care for Children in Special Schools: An Audit of Provision | Allen et al. | 2021 | Outside the United States |
| Eye Care Quality and Accessibility Improvement in the Community (EQUALITY) for Adults at Risk for Glaucoma: Study Rationale and Design | Owsley et al. | 2015 | Ineligible study design |
| Eye Care Utilization by Older Adults in Low, Middle, and High Income Countries | Vela et al. | 2012 | Outside the United States |
| Eye conditions and blindness in children: Priorities for research, programs, and policy with a focus on childhood cataract | Gilbert et al. | 2012 | Outside the United States |
| Addressing the Third Epidemic of Retinopathy of Prematurity Through Telemedicine and Technology: A Systematic Review | Al-Khaled et al. | 2021 | Outside the United States |
| Eye Health Care Utilization among Native Hawaiian and Pacific Islander Adults in the United States | Lin et al. | 2023 | Wrong population |
| Eye Health Information Systems in Selected Countries | Hashemi et al. | 2018 | Wrong population |
| Eye Health Promotion and the Prevention of Blindness in Developing Countries: Critical Issues | Hubley et al. | 2006 | Outside the United States |
| Eye Health Service Access and Utilization in the National Indigenous Eye Health Survey | Turner et al. | 2011 | Outside the United States |
| Eye Health, COVID-19, and the Occupational Health Professional: Round Table | Gallagher et al. | 2021 | Wrong area of impact |
| Eye Injury Prevention for the Pediatric Population | Hoskin et al. | 2016 | Wrong population |
| Eye Health: What Research Is Needed, and Where? | Ramke et al. | 2022 | Wrong geographic scope |
| Eyes of Understanding: Ophthalmologists' Attitudes Towards Caring for People With Intellectual Disabilities in Clinical and Nonclinical Settings | Hou et al. | 2025 | Outside the United States |
| EyeScreen: Development and Potential of a Novel Machine Learning Application to Detect Leukocoria | Bernard et al. | 2022 | Outside the United States |
| AI-Human Hybrid Workflow Enhances Teleophthalmology for the Detection of Diabetic Retinopathy | Dow et al. | 2023 | Wrong population |
| Factors Affecting Compliance with Diabetic Retinopathy Screening: A Qualitative Study Comparing English and Spanish Speakers | Hudson et al. | 2022 | Wrong population |
| Factors Affecting Global Adherence for the Uptake of Diabetic Retinopathy Screening: A Systematic Review and Meta-Analysis | Rahmati et al. | 2024 | Not specific to the United States |
| Factors influencing delayed presentation of periorbital carcinoma requiring orbital exenteration | Van Wyk et al. | 2025 | Outside the United States |
| An Approach to Assessing Patient Safety in Hospitals in Low-Income Countries | Lindfield et al. | 2015 | Outside the United States |
| Family Income and the Impact of a Children’s Health Insurance Program on Reported Need for Health Services and Unmet Health Need | Feinberg et al. | 2002 | Wrong area of impact |
| Gender Differentials in the Seeking of Eye Care | Jayaraman et al | 2013 | Outside the United States |
| Generalisability through local validation: overcoming barriers due to data disparity in healthcare | Mitchell et al. | 2021 | Wrong area of impact |
| Physician Responses to Insurance Benefit Restrictions: The Case of Ophthalmology | Abiona et al. | 2024 | Outside the United States |
| How Can We Improve the Quality of Cataract Services for All? A Global Scoping Review | Yoshizaki et al. | 2021 | Insufficient relevance to income-related or structural disparities in U.S. eye-care access. |
| How to Ensure Equitable Access to Eye Health for Children with Disabilities | Kuper et al. | 2016 | Not specific to the United States |
| Impact of the COVID-19 Pandemic on the Eye Health of Populations of Low Socioeconomic-Status: The Need for Diabetic Retinopathy Screening and Access to Spectacles | Hou et al. | 2022 | Outside the United States |
| Improvement Studies for Equitable and Evidence-Based Innovation: An Overview of the ‘IM-SEEN’ Model | Allen et al. | 2023 | Outside the United States |
| An Education-Based Intervention Investigating the Accuracy of Community-Based Optometrists Evaluating Limbal Anterior Chamber Depth | Jindal et al. | 2025 | Outside the United States |
| An Exploration of Smartphone Use by, and Support for People with Vision Impairment: A Scoping Review | Tan et al. | 2024 | Wrong area of impact |
| Indigenous Eye Health in the Americas: The Burden of Vision Impairment and Ocular Diseases | Furtado et al. | 2023 | Insufficient relevance to income-related or structural disparities in U.S. eye-care access. |
| Integrated Care Management to Improve Diabetes Outcomes in Refugee and Immigrant Patients (I-Care) | Sous et al. | 2021 | Not specific to eye care |
| Integrating Primary Care Services into a Rural Behavioral Health Facility in Northern Arizona: Perspectives of Healthcare Providers and Administrative Staff | Santos et al. | 2025 | Not specific to eye care |
| International Vision Care: Issues and Approaches | Khanna et al. | 2017 | Not specific to the United States |
| Longitudinal Analysis of Eye Health Disparities Due to Trachoma Using Country-Level Data from the Global Burden of Disease Study 2019 | Ono et al. | 2024 | Insufficient relevance to income-related or structural disparities in U.S. eye-care access. |
| The Impact of Vision Impairment on Vision-Related Quality of Life of Patients with Neovascular Age-Related Macular Degeneration | Marakis et al. | 2020 | Outside the United States |
| Evaluation of Vision Referral Program With School-Aged Children and Their Parents/Guardians | McClendon et al. | 2019 | Insufficient relevance to income-related or structural disparities in eye-care access. |
| Home-monitoring/remote optical coherence tomography in teleophthalmology in patients with eye disorders—a systematic review | Dolar-Szczasny et al. | 2024 | Insufficient relevance to income-related or structural disparities in U.S. eye-care access. |
| Primary eye health services for older adults as a component of universal health coverage: a scoping review of evidence from high income countries | Goodman et al. | 2023 | Not specific to the United States |
| Putting Health Care Where the Kids Are: US Public Attitudes About School-Based Health Centers | Haeder et al. | 2025 | Not specific to eye care disparities |
| Association of Private Equity Firm Acquisition of Ophthalmology Practices with Medicare Spending and Utilization of Ophthalmology Services | Braun et al. | 2024 | Insufficient relevance to income-related or structural disparities in eye-care access. |
| Five-Year Trends in Pediatric Vision Screening and Access in the United States | Chauhan et al. | 2023 | Insufficient relevance to income-related or structural disparities in eye-care access. |
| Relationship Between Claims-Based Frailty Index and Eye Care Utilization among Medicare Beneficiaries with Glaucoma. | Halawa et al. | 2023 | Insufficient relevance to income-related or structural disparities in eye-care access. |
| Pediatric and School-Age Vision Screening in the United States: Rationale, Components, and Future Direction | Ambrosino et al. | 2023 | Insufficient relevance to income-related or structural disparities in eye-care access. |
| Seeing Red: Associations between Historical Redlining and Present-Day Visual Impairment and Blindness | Hicks et al. | 2023 | Insufficient relevance to income-related or structural disparities in eye-care access. |
| Everything Is Connected: Social Determinants of Pediatric Health and Disease | Tarazi et al. | 2016 | Not specific to eye care |
| Sociodemographic Reporting in Artificial Intelligence Studies of Retinal Diseases: A Critical Appraisal of the Literature | Bondok et al. | 2025 | Does not examine income-related eye care access or disparities |
| Teleglaucoma: Improving Access and Efficiency for Glaucoma Care | Kassam et al. | 2013 | Outside the United States |
| Strengthening Eye Health Evidence for Children in Low-Income and Middle-Income Countries | Ramke et al. | 2018 | Outside the United States |
| Telemedicine and Ocular Health in Diabetes Mellitus | Bursell et al. | 2012 | Not specific to U.S. low-income populations |
| The Association of Lapses in Diabetic Retinopathy Care with Vision Impairment | Zhu et al. | 2025 | Insufficient relevance to income-related or structural disparities in eye-care access. |
| The Better Operative Outcomes Software Tool (BOOST) Prospective Study: Improving the Quality of Cataract Surgery Outcomes in Low-Resource Settings | McGuinness et al. | 2025 | Outside the United States |
| The burden of acute eye conditions on different healthcare providers: a retrospective population-based study | Rawlings et al. | 2024 | Outside the United States |
| The challenges of providing eye care for adults with intellectual disabilities | Li et al. | 2015 | Outside the United States |
| The clinical effectiveness of telehealth: A systematic review of meta-analyses from 2010 to 2019 | Snoswell et al. | 2023 | Not specific to eye care disparities |
| The Economic Burden of Vision Loss and Eye Disorders among the United States Population Younger than 40 Years | Wittenborn et al. | 2013 | Insufficient relevance to income-related or structural disparities in eye-care access. |
| The Evidence on School-Based Health Centers: A Review | Arenson et al. | 2019 | Not specific to eye care |
| The Growth of Private Equity Investment in Health Care: Perspectives from Ophthalmology | O’Donnell et al. | 2020 | Insufficient relevance to income-related or structural disparities in eye-care access. |
| Utility Index and Vision-Related Quality of Life in Patients Awaiting Specialist Eye Care | Araujo et al. | 2024 | Outside the United States |
| Designing Hierarchical Networks to Improve Patient Access to Care: A Case Study in Veteran Eye Care | VanDeusen et al. | 2021 | Insufficient relevance to income-related or structural disparities in eye-care access. |
| A Blurry Bottom Line: The Economics of Declining Medicare Payment Trends in Ophthalmology Subspecialties, 2003 to 2024 | Bhatt et al. | 2025 | Insufficient relevance to income-related or structural disparities in eye-care access. |
| Access to Primary Eye Care for People Living with Dementia: A Call to Action for Primary Care Practitioners to ‘Think Vision’ | Piano et al. | 2024 | Outside the United States |
| Trends in Medicare Submitted Charges to Allowed Payment Ratios for Ophthalmology Services | Elhusseiny et al. | 2024 | Insufficient relevance to income-related or structural disparities in eye-care access. |
| Annual Diabetic Eye Examinations in a Managed Care Medicaid Population | Hatef et al. | 2015 | Focused on disease-specific diabetic retinopathy screening rather than general eye care access |
| Applying RE-AIM to Evaluate Two Community-Based Programs Designed to Improve Access to Eye Care for Those at High-Risk for Glaucoma | Sapru et al. | 2017 | Evaluates glaucoma specific detection programs rather than general eye care |
| Between Scylla and Charybdis?—Health Insurance Claims-Data to Monitor Quality of Service Delivery in Ophthalmology | Schmid et al. | 2024 | Insufficient relevance to income-related or structural disparities in eye-care access. |
| A Novel Optometry-Led Decision-Making Community Referral Refinement Scheme for Neovascular Age-Related Macular Degeneration Screening | Sanders et al. | 2024 | Conducted outside the United States |
| An Updated Estimate of Costs of Endophthalmitis Following Cataract Surgery Among Medicare Patients: 2010–2014 | Schmier et al. | 2016 | Focuses on costs of cataract surgery complications rather than eye care access disparities |
| Analysis of an International Collaboration for Capacity Building of Human Resources for Eye Care: Case Study of the College-College VISION 2020 LINK | Mwangi et al. | 2017 | Conducted outside the United States |
| Analysis of hesitancy and motivational factors for COVID-19 vaccination among patients presenting to eye care hospitals – A multicenter questionnaire-based survey | Kaur et al. | 2022 | Conducted outside the United States |
| AOA Ophthalmology and Otolaryngology Program Closures as a Model to Highlight Challenges of Maintaining GME in High Need Areas | Ahmed et al. | 2022 | Not relevant to patient level eye care disparities |
| Apple Vision Pro for Ophthalmology and Medicine | Masalkhi et al | 2023 | Not relevant to disparities in eye care access |
| Application of Artificial Intelligence Models to Predict the Onset or Recurrence of Neovascular Age-Related Macular Degeneration | Sorrentino et al. | 2024 | Not relevant to disparities in eye-care access |
| Application of Telemedicine System on the Management of General Patient in Quarantine | Lu et al. | 2023 | Not specific to eye care and conducted outside the United States |
| Are We Advancing Universal Health Coverage Through Cataract Services? Protocol for a Scoping Review | Lee et al. | 2020 | Study protocol with no reported findings |
| Mitigation of AI Adoption Bias Through an Improved Autonomous AI System for Diabetic Retinal Disease | Abràmoff et al. | 2024 | Disease specific rather than general eye care |
| Patient Perceived Value of Teleophthalmology in an Urban, Low Income US Population with Diabetes | Ramchandran et al. | 2020 | Disease specific rather than general eye care |

|  |  |  |  |  |  |
| --- | --- | --- | --- | --- | --- |
| **Supplementary Table S5. Rationale for Inclusion and Contribution of Grey Literature Sources Identified Outside the Database Search** | | | | | |
| Title | Source | Year of Publication | Document Type | Reason for Inclusion | Contribution to Review |
| Community Health Centers in an Era of Health System Reform and Economic Downturn: Prospects and Challenges | George Washington University Health Policy & Management Faculty Publications (prepared by Rosenbaum S. et al.) | 2009 | Policy Report | Included to provide policy context on structural factors influencing health care access for underserved populations | Provided evidence on how health system capacity, geographic variation, and community health center infrastructure influence access to care |
| H.R. 5304 – Departments of Labor, Health and Human Services, and Education, and Related Agencies Appropriations Act, 2026 | U.S. Congress (Congress.gov) | 2025 | Federal legislative document | Included to provide contemporary legislative context on federal health care funding and policy priorities relevant to eye care. | Provided evidence on federal funding priorities, including proposed reductions to the Vision Health Initiative and continued funding for the National Eye Institute |

| **Supplementary Table S6. Characteristics and Key Findings of Sources Identified Through the Database Search (Complete Abstracted Data)** | | | | | | | |
| --- | --- | --- | --- | --- | --- | --- | --- |
| **Title** | **Author** | **Year** | **Study Type** | **Study Setting** | **Study Population** | **Methodological Approach** | **Key Findings** |
| Socioeconomic disparity in use of eye care services among US adults with age-related eye diseases: National Health Interview Survey, 2002 and 2008. | Zhang X, Beckles GL, Chou C-F, Saaddine JB, Wilson MR, Lee PP, Parvathy N, Ryskulova A, Geiss LS. | 2013 | Repeated cross-sectional study | United States | U.S. adults ≥40 years with self-reported age-related eye disease | Secondary analysis of 2002 and 2008 NHIS data | Persistent socioeconomic disparities were observed in eye care access and utilization. Visual impairment has been associated with depression, loss of productivity, injuries, and premature death |
| Eye Care Among US Adults at High Risk for Vision Loss in the United States in 2002 and 2017 | Saydah SH, Gerzoff RB,  Saaddine JB, Zhang X, Cotch MF | 2020 | Repeated cross-sectional study | United States | U.S. adults ≥18 years, including adults at high risk for vision loss | Secondary analysis of nationally representative National Health Interview Survey (NHIS) data from 2002 and 2017 | 9% of people who reported a need for glasses in 2017 also reported not being able to afford them |
| Disparities in vision impairment among adults in the United States. | Wilson CJ, Rust G, Levine R, Alema-Mensah E. | 2008 | Cross-sectional observational study | United States | U.S. adults aged ≥20 years who participated in NHANES 2001–2002 | Secondary analysis of National Health and Nutrition Examination Survey (NHANES) visual acuity measurements to examine disparities in vision impairment | Higher household income (>$75,000) was associated with lower odds of visual impairment |
| Disparities in Vision Health and Eye Care | Elam AR, Tseng VL, Rodríguez TM, Mike EV, Warren AK, Coleman AL. | 2022 | Narrative review | United States | Not applicable; no single study population | Narrative synthesis of existing literature on vision health and eye-care disparities | Children whose  families who are below the federal poverty line are twice as likely to have visual impairment as children in families 200% above the federal poverty line |
| Most State Medicaid Programs Cover Routine Eye Exams for Adults, But Coverage of Other Routine Vision Services Varies. | Lipton BJ, Garcia J, Boudreaux MH, Azatyan P, McInerney MP | 2024 | Cross-sectional policy analysis | Varying states around the U.S. | U.S. state Medicaid programs and adult vision benefit policies | State-level analysis of Medicaid adult vision coverage policies | More than 6 million Medicaid enrollees reside in states lacking comprehensive vision exam coverage, and more than 14 million in states lacking coverage for eyeglasses |
| Comparison of Access to Eye Care Appointments Between Patients with Medicaid and Those with Private Health Care Insurance. | Lee YH, Chen AX, Varadaraj V, Hong GH, Chen Y, Friedman DS, Stein JD, Kourgialis N, Ehrlich JR. | 2018 | Prospective cohort study | Eye-care practices in Michigan and Maryland | 330 randomly selected ophthalmologists and optometrists | Telephone-based comparison of appointment access by insurance type | It is more difficult to obtain an eye care appointment for individuals with Medicaid than for those with private insurance |
| Assessing the need for on-site eye care professionals in community health centers. | Shin P, Finnegan B | 2009 | Cross-sectional survey study | U.S. federally qualified health centers | 100 federally qualified health centers | Electronic survey of a random sample of federally qualified health centers | Nearly 7 out of 10 FQHC patients have incomes at or below the federal poverty level. But 69% of FQHCs do not provide on-site comprehensive eye exams |
| Sociodemographic Factors Associated with Emergent Eye-Related Emergency Department Visits: A Multicenter Analysis. | Quintero M, Mahjoub H, Ssekasanvu J, Yonekawa Y, Justin GA, Cavuoto KM, Lorch A, Madan V, Sivakumar I, Zhao X, et al. | 2025 | Cross-sectional multicenter study | Four U.S. academic eye-care emergency departments | 60,677 patients with eye-related ED visits | Multicenter analysis of eye-related ED encounters and sociodemographic factors | Uninsured populations rely more heavily on emergency departments (EDs) for eye-related concerns. Lack of insurance coverage is associated with emergent eye-related cases in the ED. Access to ophthalmologic care in uninsured communities can reduce the pipeline of untreated chronic disease to ED eye emergency cases |
| Public Attitudes About Eye and Vision Health. | Scott AW, Bressler NM, Ffolkes S, Wittenborn JS, Jorkasky J. | 2016 | Cross-sectional survey study | United States | 2,044 U.S. adults | Online survey assessing attitudes and awareness of eye health | 25% of participants were unfamiliar with any eye conditions, 37.3% had never heard of diabetic retinopathy, and half did not know of the existence of macular degeneration |
| The Importance of Health Literacy in Addressing Eye Health and Eye Care Disparities. | Capó, H., Edmond, J. C., Alabiad, C. R., Ross, A. G., Williams, B. K., & Briceño, C. A. | 2022 | Literature review | United States | U.S.-based studies on eye health literacy and disparities | Retrospective literature review using PubMed and citation searching | Low health literacy is a strong indicator of appointment absence and low usage of medication in patients with glaucoma |
| Disparities in delivery of ophthalmic care; An exploration of public Medicare data. | Lee CS, Su GL, Baughman DM, Wu Y, Lee AY. | 2017 | Cross-sectional study | United States | 2.2 million Medicare beneficiaries who underwent cataract surgery | Analysis of Medicare cataract surgery data using geographic and statistical modeling | Cataract care recipients  who resided in rural areas traveled  further for ophthalmic care than urban and suburban  residents |
| Disparities in access to medical care for individuals with vision impairment. | Spencer C, Frick K, Gower EW, Kempen JH, Wolff JL. | 2009 | Cross-sectional study | United States | 40,643 noninstitutionalized U.S. adults aged >40 years | Secondary analysis of pooled 2002–2004 MEPS data | Individuals with visual impairment were more than twice as likely to rely on  Medicaid than individuals without |
| Demographic and Socioeconomic Differences in Outpatient Ophthalmology Utilization in the United States. | Rasendran C, Tye G, Knusel K, Singh RP. | 2020 | Retrospective cross-sectional study | U.S. outpatient ophthalmology care | U.S. adults with outpatient ophthalmology visits | Analysis of nationally representative healthcare utilization data | Individuals with lower income levels report a greater prevalence of diabetic  retinopathy than their counterparts |
| Making Eye Health a Population Health Imperative: Vision for Tomorrow. | National Academies of Sciences, Engineering, and Medicine, Health and Medicine Division, Board on Population Health and Public Health Practice, Committee on Public Health Approaches to Reduce Vision Impairment and Promote Eye Health. | 2016 | Consensus report/evidence review | United States | Not Applicable; Population-level evidence synthesis | Committee review and synthesis of evidence on vision and eye health | The National Academies of Sciences, Engineering, and Medicine have highlighted vision health as a population  health priority |
| Outcomes of a vision screening program for underserved populations in the United States. | Friedman DS, Cassard SD, Williams SK, Baldonado K, O’Brien RW, Gower EW. | 2013 | Program evaluation study | U.S. free health clinic | 3,004 free health clinic patients receiving vision screening | Vision screening with visual acuity testing and questionnaire followed by referral for eye examination | Majority of uninsured adults in the program showed high rates of visual problems that required more comprehensive treatment, yet rates of follow-up with an eye care professional after referral remained consistently low |
| Essential Health Benefits and the Affordable Care Act: Law and Process. | Bagley N, Levy H. | 2014 | Policy and legal analysis | United States | Not Applicable | Case study analysis of ACA essential health benefits policy and implementation | Policy measures, including provisions in the Affordable Care Act, have expanded vision coverage for pediatric populations; however, coverage for adult vision services is not uniformly mandated |
| Understanding the Knowledge Gap Experienced by U.S. Safety Net Patients in Teleretinal  Screening | George SM, Hayes EM, Fish A, Daskivich LP, Ogunyemi OI. | 2017 | Qualitative focus group study | Six urban safety-net primary care clinics in South Los Angeles, California | 42 African American and Latino adults with diabetes who received teleretinal screening | Six focus groups analyzed using modified grounded theory | Teleophthalmology enables more patients in underserved communities to connect with vision care providers, but challenges related to health literacy and patient engagement persist |

| **Supplementary Table S7: Critical Appraisal of Analytical Cross-Sectional Studies** | | | | | | | | | | |
| --- | --- | --- | --- | --- | --- | --- | --- | --- | --- | --- |
| **Title** | **Author** | **Year** | **Q1** | **Q2** | **Q3** | **Q4** | **Q5** | **Q6** | **Q7** | **Q8** |
| Socioeconomic disparity in use of eye care services among US adults with age-related eye diseases: National Health Interview Survey, 2002 and 2008. | Zhang X, Beckles GL, Chou C-F, Saaddine JB, Wilson MR, Lee PP, Parvathy N, Ryskulova A, Geiss LS. | 2013 | Yes, U.S. adults ≥40 years old with self-reported age-related eye disease | No. ARED status was self-reported | Yes, socioeconomic position measured through poverty-income ratio | Yes, however self-reported answers are vulnerable to bias | Yes, confounding variables including age, sex, race, and health insurance status | Yes, multivariable logistic regression was adjusted for confounding variables | Yes, multivariable regression and inequality analyses accounted for NHIS survey design | Yes, NHIS survey is described in detail |
| Eye Care Among US Adults at High Risk for Vision Loss in the United States in 2002 and 2017. | Saydah SH, Gerzoff RB, Saaddine JB, Zhang X, Cotch MF | 2020 | Yes, NHIS adults ≥18 years clearly defined as high risk for vision loss | No, high-risk status labeled through self-reporting | Yes, standardized NHIS measures for demographic and socioeconomic variables were used | No, three main outcomes were self-reported | Yes, confounding variables including age, sex, marital status, etc. were identified | Yes, multivariable logistic regression models were used to evaluate associations | Yes, complex NHIS sampling design was accounted for | Yes, NHIS survey is described in-detail |
| Disparities in vision impairment among adults in the United States. | Wilson CJ, Rust G, Levine R, Alema-Mensah E. | 2008 | Yes, analysis was limited to adults 18–85 years with available visual-acuity data | Yes, visual impairment and acuity parameters were clearly defined | Yes, standardized NHANES racial and ethnic classifications were used | Yes, vision impairment was objectively measured | Yes, confounding variables including age, income, and educational level were identified | Yes, multiple logistic regression was used to account for potential confounding variables | Yes, bivariate analyses and multivariable logistic regression were used to account | Yes, participant characteristics and the nationally representative U.S. NHANES setting were clearly described |
| Sociodemographic Factors Associated with Emergent Eye-Related Emergency Department Visits: A Multicenter Analysis. | Quintero M, Mahjoub H, Ssekasanvu J, Yonekawa Y, Justin GA, Cavuoto KM, Lorch A, Madan V, Sivakumar I, Zhao X, et al. | 2025 | Yes, eye-related concerns who visited the four specified EDs during Jan–Dec 2019 | Yes, diagnoses based on clinical diagnoses | Yes, sociodemographic exposures were gathered from patient encounters | Yes, primary outcome determined from clinical diagnoses | Yes, confounding variables including sex, race, insurance, age, and ED setting were identified | Yes, analysis to evaluate associations were used | Yes, statistical comparisons and regression analyses were used to examine associations | Yes, 60,677 patients with eye-related ED concerns and 4 ED settings were clearly defined |
| Disparities in delivery of ophthalmic care: An exploration of public Medicare data. | Lee CS, Su GL, Baughman DM, Wu Y, Lee AY. | 2017 | Yes, 2012 Medicare cataract surgeries were identified using specified CPT codes and defined data sources. | Yes, Medicare cataract patients, U.S. geographic setting, and study period were clearly described. | Yes, access was objectively measured using provider locations, geocoding, and calculated distance to providers | Yes, cataract surgeries were identified using standardized CPT procedure codes in Medicare claims data | Yes, confounding factors including age, race, and sex were identified | Yes, age was accounted for using age-specific cataract surgery estimates. However, race and sex could not be adjusted for | Cataract surgery utilization and geographic access were measured using Medicare claims, Census data, and defined geographic methods | Regression modeling and geographic analyses were used to compare cataract surgery delivery |
| Disparities in access to medical care for individuals with vision impairment. | Spencer C, Frick K, Gower EW, Kempen JH, Wolff JL. | 2009 | Yes, sample was clearly defined as MEPS respondents aged ≥40 years, categorized by self-reported blindness, vision impairment, or no vision impairment. | No, blindness and vision impairment were self-reported | No, Vision status was measured using self-reported responses to MEPS vision questions rather than objective vision testing | Yes, access to care was measured using defined MEPS measures of usual source of care, unmet need, and delays or inability to obtain care. | Yes, confounders included sex, race, age, marital status, family size, education, income, etc. were identified | Yes, logistic regression adjusted for potentially confounding variables | Yes, weighted analyses and logistic regression accounting for the complex MEPS survey design | Yes, study population and setting were clearly described |
| Demographic and Socioeconomic Differences in Outpatient Ophthalmology Utilization in the United States. | Rasendran C, Tye G, Knusel K, Singh RP. | 2020 | Yes, MEPS respondents from 2007–2015 and identification of participants with ophthalmologic conditions | No, ophthalmologic conditions were self-reported | Yes, demographic and socioeconomic exposures were defined using standardized MEPS variables | Yes, outpatient ophthalmologic utilization was measured using MEPS health care utilization data | Yes, confounding variables including race, income, insurance, education, and geographic region were identified | Yes, multivariable analyses adjusted for patient characteristics | Yes, adjusted regression analyses were used to evaluate associations | Yes, the study described 183,054 nationally representative, noninstitutionalized U.S. MEPS respondents from 2007–2015 |

| **Supplementary Table S8: Critical Appraisal of Descriptive Cross-Sectional Studies** | | | | | | | | | | | | | |
| --- | --- | --- | --- | --- | --- | --- | --- | --- | --- | --- | --- | --- | --- |
| **Title** | **Author** | **Year** | **Q1** | **Q2** | **Q3** | **Q4** | **Q5** | **Q6** | **Q7** | **Q8** | **Q9** | **Q10** | **Q11** |
| Assessing the need for on-site eye care professionals in community health centers. | Shin P, Finnegan B | 2009 | Yes, the study examined on-site eye care capacity and barriers among U.S. community health centers | Yes, A cross-sectional survey was appropriate to assess current eye care capacity and barriers | Yes, A random sample of 300 federally qualified health centers was selected nationwide | No, survey measures were described, but validation or reliability of the survey was not reported | Yes, an electronic survey directly assessed eye care services, capacity, and barriers | Unsure. 100 FQHCs responded; however, a sample size calculation was not done | Results were represented as proportions and displayed that most FQHCs do not have on-site comprehensive eye care | Yes, results were weighted for nonresponse. However, some comparisons lacked statistical testing | Yes, findings clearly identified limited on-site eye care | Yes, however 33% response rates limit generalizability | The study identifies important barriers and opportunities for expanding eye care in underserved communities. |
| Public Attitudes About Eye and Vision Health. | Scott AW, Bressler NM, Ffolkes S, Wittenborn JS, Jorkasky J. | 2016 | Yes, the study examined attitudes and awareness of eye health among U.S. adults across racial groups | Yes, a nationwide cross-sectional survey was appropriate for assessing public attitudes and awareness | Yes, participants were randomly selected from diverse nationwide online panels with minority oversampling. | No formal validity testing and pilot testing were performed | Yes, the survey directly measured attitudes and awareness | Yes, the sample size of 2,044 and minority oversampling provided sufficiently precise estimates | Results were clearly reported using weighted percentages and 95% confidence intervals | Yes, sampling weights and confidence intervals were used to account for the complex sampling design | Yes, high value placed on vision but gaps in eye disease and risk-factor awareness | Yes, broadly representative of the U.S. but online and English-only recruitment may limit generalizability | The study identifies important gaps in eye-health awareness and informs public education and research priorities. |
| Most State Medicaid Programs Cover Routine Eye Exams for Adults, But Coverage of Other Routine Vision Services Varies. | Lipton BJ, Garcia J, Boudreaux MH, Azatyan P, McInerney MP | 2024 | Yes, the study examines Medicaid coverage of routine vision services for adults across U.S. states | Yes, a cross-sectional analysis was appropriate for comparing Medicaid vision coverage across states | Yes, state Medicaid programs were comprehensively included with clear criteria for exclusions | Yes, coverage was assessed using official sources and independently reviewed by multiple authors | Yes, vision coverage data were collected from official Medicaid sources, with agencies and plans contacted when information was unclear | Yes, the analysis included Medicaid coverage policies across all 50 states and D.C. | Results are presented as state counts, percentages, and enrollee estimates with the main finding being substantial gaps in Medicaid coverage for eyeglasses | Yes, coverage was systematically categorized and independently assessed with discrepancies resolved | Yes, the findings clearly identify state-level gaps and variation in Medicaid vision coverage | Yes, findings are broadly applicable to U.S. Medicaid populations and state policy contexts | The study identifies important gaps in Medicaid vision coverage and opportunities to improve access to routine eye care |

| **Supplementary Table S9: Critical Appraisal of Cohort Study** | | | | | | | | | | | | | |
| --- | --- | --- | --- | --- | --- | --- | --- | --- | --- | --- | --- | --- | --- |
| **Title** | **Author** | **Year** | **Q1** | **Q2** | **Q3** | **Q4** | **Q5** | **Q6** | **Q7** | **Q8** | **Q9** | **Q10** | **Q11** |
| Comparison of Access to Eye Care Appointments Between Patients with Medicaid and Those with Private Health Care Insurance. | Lee YH, Chen AX, Varadaraj V, Hong GH, Chen Y, Friedman DS, Stein JD, Kourgialis N, Ehrlich JR. | 2018 | Yes, identical practices were contacted using standardized Medicaid and private insurance scenarios | Yes, insurance status was consistently defined as Medicaid or BCBS private insurance | Yes, insurance type was explicitly assigned and standardized in each simulated call | Yes, the study design accounted for patient scenario, practice characteristics, location, and specialty | Yes, standardized scripts and statistical modeling were stated | Yes, appointment outcomes had not occurred before each simulated appointment request | Yes, appointment success and wait time were objectively recorded | Yes, appointment success and wait time were determined during each simulated appointment request | Yes, appointment outcomes were recorded for the simulated requests, with no meaningful loss to follow-up reported | Not applicable, longitudinal follow-up was not required. | Yes, appropriate regression analyses were used to compare appointment access by insurance type |

| **Supplementary Table S10: Critical Appraisal of Qualitative Study** | | | | | | | | | | | | |
| --- | --- | --- | --- | --- | --- | --- | --- | --- | --- | --- | --- | --- |
| **Title** | **Author** | **Year** | **Q1** | **Q2** | **Q3** | **Q4** | **Q5** | **Q6** | **Q7** | **Q8** | **Q9** | **Q10** |
| Understanding the Knowledge Gap Experienced by U.S. Safety Net Patients in Teleretinal  Screening | George SM, Hayes EM, Fish A, Daskivich LP, Ogunyemi OI. | 2017 | Yes, modified grounded theory aligned with exploring patient experiences | Yes, focus-group methodology was used to understand safety-net patient experience and knowledge gap in teleretinal screening | Yes, six 90-minute focus groups with 42 African American and Latino patients were used to collect experiential data | Yes, transcripts were systematically coded and analyzed using modified grounded theory techniques | Yes, interpretations were developed from identified themes regarding patient experiences | No, researchers did not explicitly describe their cultural or theoretical positioning | No, researcher influence was not explicitly addressed | Yes, participant characteristics and direct quotations were provided throughout the findings | Unsure, participant consent is reported but formal ethics approval is not clearly stated | Yes, conclusions about knowledge gaps were supported by identified themes and participant experiences |

| **Supplementary Table S11: Critical Appraisal of Expert Opinion** | | | | | | | | |
| --- | --- | --- | --- | --- | --- | --- | --- | --- |
| **Title** | **Author** | **Year** | **Q1** | **Q2** | **Q3** | **Q4** | **Q5** | **Q6** |
| Making Eye Health a Population Health Imperative: Vision for Tomorrow. | National Academies of Sciences, Engineering, and Medicine, Health and Medicine Division, Board on Population Health and Public Health Practice, Committee on Public Health Approaches to Reduce Vision Impairment and Promote Eye Health. | 2016 | Yes, expert committee members, professional roles, and affiliations are clearly identified | Yes, the committee includes experts in eye care, public health, epidemiology, and health policy | Yes, the report focuses on improving eye health and access to care, particularly for underserved populations | Yes, conclusions and recommendations are supported by research, expert input, and committee analysis | Yes, extensive existing research and evidence are cited throughout the report | Yes, conflicting evidence and research gaps are acknowledged and discussed |

| **Supplementary Table S12: Critical Appraisal of Narrative Reviews** | | | | | | | | | | | |
| --- | --- | --- | --- | --- | --- | --- | --- | --- | --- | --- | --- |
| **Title** | **Author** | **Year** | | **1** | **2** | **3** | **4** | **5** | **6** | | **Total** |
| Disparities in Vision Health and Eye Care | Elam AR, Tseng VL, Rodriguez TM, Mike EV, Warren AK, Coleman AL. | 2022 | 2/2 Author clearly establishes the significance and persistence of disparities in vision health and eye care | | 2/2 Author clearly states aim to describe vision health disparities and examine contributing factors | 0/2 Literature search methods, databases, and search terms were not described | 2/2  Key statements are consistently supported by relevant sources | 2/2 Conclusions and recommendations are supported by evidence from the reviewed literature | 2/2 Relevant data from the reviewed studies are clearly presented to support key points | 10/12 | |
| The Importance of Health Literacy in Addressing Eye Health and Eye Care Disparities. | Capó, H., Edmond, J. C., Alabiad, C. R., Ross, A. G., Williams, B. K., & Briceño, C. A. | 2022 | 2/2 Authors clearly establish the importance of health literacy in addressing eye health and eye care disparities | | 2/2 Authors clearly state aims to evaluate health literacy in eye care and explore strategies to reduce disparities | 2/2 Authors provide the databases searched, search terms, and inclusion/exclusion criteria | 2/2 Key statements are consistently supported by relevant sources | 2/2 Conclusions and recommendations are supported by evidence from the reviewed literature | 2/2 Relevant data from the reviewed studies are clearly presented to support key points | 12/12 | |
| Essential Health Benefits and the Affordable Care Act: Law and Process. | Bagley N, Levy H. | 2014 | 2/2 Authors clearly establish the importance of essential health benefits for insurance coverage and ACA implementation | | 2/2 Authors clearly state two questions addressing the legality and administrative process of HHS’s essential health benefits policy | 1/2 Authors use extensive legal, government, and academic sources but do not describe a formal literature search strategy | 2/2 Key statements are consistently supported by relevant sources | 2/2 Conclusions and recommendations are supported by evidence from the reviewed literature | 1/2 Some relevant quantitative data are presented, but detailed outcome data are limited | 10/12 | |

| **Supplementary Table S13: Critical Appraisal of Program Evaluation** | | | | | | | | | | | | |
| --- | --- | --- | --- | --- | --- | --- | --- | --- | --- | --- | --- | --- |
| **Title** | **Author** | **Year** | **Q1** | **Q2** | **Q3** | **Q4** | **Q5** | **Q6** | **Q7** | **Q8** | **Q9** | **Q10** |
| Outcomes of a vision screening program for underserved populations in the United States. | Friedman DS, Cassard SD, Williams SK, Baldonado K, O’Brien RW, and Gower EW. | 2013 | Unsure, patients were screened on specific clinic days, but consecutive or random enrollment was not clearly reported | Yes, participants were screened without being selected based on known eye disease status | Yes, no inappropriate exclusions based on screening results or eye disease status were reported | Yes, Vision screening was completed before the professional eye exam therefore, results were not yet known | Yes, Referral thresholds were predefined, including visual acuity of 20/50 or worse and specified questionnaire criteria | Yes, a comprehensive eye exam by an optometrist or ophthalmologist was used to identify eye conditions | Unsure, the study does not report whether eye examiners were blinded to the screening results | Unsure, the time between screening and the professional eye exam varied | Yes, participants who completed follow-up received the same comprehensive professional eye examination | No, less than half of referred screen-positive participants completed the professional eye exam |

| **Supplementary Table S14: Critical Appraisal of Grey Literature** | | | | | | | | |
| --- | --- | --- | --- | --- | --- | --- | --- | --- |
| **Title** | **Author** | **Year** | **Q1** | **Q2** | **Q3** | **Q4** | **Q5** | **Q6** |
| Community Health Centers in an Era of Health System Reform and Economic Downturn: Prospects and Challenges. | Rosenbaum S, Finnegan B, Shin P. | 2009 | Yes, the report was authored by experienced health policy researchers and published by a reputable health policy organization | Yes, the report has a clearly stated purpose and supports its analysis with UDS data, government sources, legislation, and published research | Yes, the report clearly focuses on U.S. community health centers and their role and challenges within the changing health care and economic environment | Yes, the report presents opportunities offered and challenges experienced by health centers | Yes, the report is clearly dated March 2009 and incorporates contemporary data, legislation, and policy developments relevant to that period | Yes, the report highlights how policy and funding challenges influence community health centers and the care they provide to underserved populations |

| **Supplementary Table S15. Coding of Included Sources Using Kingdon's Multiple Streams Framework** | | | | | |
| --- | --- | --- | --- | --- | --- |
| **Title** | **Author** | **Problem** | **Policy** | **Politics** | **Relevant Evidence** |
| Socioeconomic Disparity in Use of Eye Care Services Among US Adults With Age-Related Eye Diseases: National Health Interview Survey, 2002 and 2008 | Zhang et al. | ✓ |  |  | Lower socioeconomic position was associated with lower eye care utilization, and socioeconomic disparities persisted from 2002 to 2008 |
| Eye Care Among US Adults at High Risk for Vision Loss in the United States in 2002 and 2017 | Saydah et al. | ✓ |  |  | Income-related disparities in eye care persisted from 2002 to 2017, with lower-income adults reporting lower eye care use and greater difficulty affording eyeglasses |
| Disparities in Vision Impairment Among Adults in the United States | Wilson et al. | ✓ |  |  | Higher household income was associated with lower odds of vision impairment, with income >$75,000 showing a protective association compared with income <$25,000 |
| Disparities in Vision Health and Eye Care | Elam et al. | ✓ | ✓ |  | Lower income was associated with greater vision impairment and reduced eye care utilization, proposed strategies included expanding insurance coverage, FQHC-based care, and teleophthalmology |
| Most State Medicaid Programs Cover Routine Eye Exams for Adults, But Coverage of Other Routine Vision Services Varies | Lipton et al. | ✓ | ✓ | ✓ | State Medicaid policies varied substantially in adult vision coverage, leaving millions of enrollees without comprehensive eye exam or eyeglass coverage |
| Comparison of Access to Eye Care Appointments Between Patients With Medicaid and Those With Private Health Care Insurance | Lee et al. | ✓ | ✓ | ✓ | Medicaid patients were less likely to obtain eye care appointments than privately insured patients, increased Medicaid reimbursement and improved care coordination as solutions |
| Assessing the Need for On-Site Eye Care Professionals in Community Health Centers | Shin et al. | ✓ | ✓ | ✓ | Most community health centers lacked on-site comprehensive eye care, cost and inadequate reimbursement identified as major barriers, strategies included financial support and partnerships to expand eye care services |
| Sociodemographic Factors Associated With Emergent Eye-Related Emergency Department Visits: A Multicenter Analysis | Quintero et al. | ✓ | ✓ |  | Uninsured patients were more likely to present with emergent eye conditions, redirecting nonemergent cases to outpatient clinics identified as potential solution |
| Public Attitudes About Eye and Vision Health | Scott et al. | ✓ | ✓ | ✓ | Insurance limited eye exam frequency for one-third of respondents, most supported eye care research, while nearly half considered federal research funding insufficient |
| The Importance of Health Literacy in Addressing Eye Health and Eye Care Disparities | Capo et al. | ✓ | ✓ |  | Low health literacy and socioeconomic barriers contribute to reduced eye care use, targeted education and accessible health information identified to reduce disparities |
| Disparities in delivery of ophthalmic care; An exploration of public Medicare data | Lee et al. | ✓ | ✓ |  | Geographic disparities in cataract care were associated with greater travel distances and lower provider availability, resource allocation and provider distribution were identified as potential responses |
| Disparities in Access to Medical Care for Individuals with Vision Impairment | Spencer et al. | ✓ | ✓ | ✓ | Lower income and financial barriers were associated with access problems, expanded eye care coverage and accessible transportation were proposed, with political action identified as necessary for implementation. |
| Demographic and Socioeconomic Differences in Outpatient Ophthalmology Utilization in the United State | Rasendran et al. | ✓ | ✓ |  | Lower income and lack of private insurance were associated with reduced outpatient ophthalmology use, increased access to eye care providers was proposed to address these disparities |
| Making Eye Health a Population Health Imperative: Vision for Tomorrow (2016) | National Academies of Sciences, Engineering, and Medicine | ✓ | ✓ | ✓ | Socioeconomic and coverage barriers contribute to inequities in eye care, expanded clinical access, stronger public health capacity, and coordinated federal and state action were identified as responses |
| Outcomes of a Vision Screening Program for Underserved Populations in the United State | Friedman et al. | ✓ | ✓ |  | Underserved adults had substantial unmet eye care needs and low follow up after referral, immediate, on-site eye examinations were proposed to improve follow up |
| Essential Health Benefits and the Affordable Care Act: Law and Process | Bagley et al. |  | ✓ | ✓ | ACA essential health benefits were implemented through state-selected benchmark plans, with political pressures, stakeholder input, and state–federal dynamics shaping implementation |
| Understanding the Knowledge Gap Experienced by U.S. Safety Net Patients  in Teleretinal Screenin | George et al. | ✓ | ✓ |  | Socioeconomic, health literacy, and technology related barriers created gaps in patient understanding of teleretinal screening, improved patient education and provider communication were proposed to address these barriers. |
| Community Health Centers in an Era of Health System Reform and Economic Downturn: Prospects and Challenges | Rosenbaum et al. | ✓ | ✓ | ✓ | Community health centers serve large numbers of low-income and uninsured patients facing financial, social, and geographic barriers to care. Their capacity to expand access is strongly influenced by Medicaid financing, federal grants, workforce investment, and broader health reform policies. |
| H.R. 5304, Departments of Labor, Health and Human Services, and Education, and Related Agencies Appropriations Act, 2026 | U.S. Congress |  | ✓ | ✓ | Federal appropriations demonstrate the role of governmental funding priorities in sustaining health and vision programs, including $896.5 million proposed for the National Eye Institute and substantial federal funding for Medicaid, illustrating how congressional budget decisions shape the policy environment for eye care access. |
